# Projective quasi-synchronization of coupled memristive neural networks with uncertainties and impulsive effect

**DOI:** 10.3389/fnbot.2022.985312

**Published:** 2022-09-09

**Authors:** Manman Yuan, Xiong Luo, Jun Hu, Songxin Wang

**Affiliations:** ^1^School of Computer and Communication Engineering, University of Science and Technology Beijing, Beijing, China; ^2^Shunde Graduate School, University of Science and Technology Beijing, Foshan, China; ^3^Beijing Key Laboratory of Knowledge Engineering for Materials Science, Beijing, China; ^4^School of Economics and Management, Fuzhou University, Fuzhou, China; ^5^School of Information Management and Engineering, Shanghai University of Finance and Economics, Shanghai, China

**Keywords:** event-triggered, memristor, coupled neural networks, projective quasi-synchronization, uncertainties

## Abstract

The dynamic behavior of memristive neural networks (MNNs), including synchronization, effectively keeps the robotic stability against numerous uncertainties from the mimic of the human brain. However, it is challenging to perform projective quasi-synchronization of coupled MNNs with low-consumer control devices. This is partly because complete synchronization is difficult to realize under various projective factors and parameter mismatch. This article aims to investigate projective quasi-synchronization from the perspective of the controller. Here, two approaches are considered to find the event-triggered scheme for lag synchronization of coupled MNNs. In the first approach, the projective quasi-synchronization issue is formulated for coupled MNNs for the first time, where the networks are combined with time-varying delays and uncertainties under the constraints imposed by the frequency of controller updates within limited system communication resources. It is shown that our methods can avoid the Zeno-behavior under the newly determined triggered functions. In the second approach, following classical methods, a novel projective quasi-synchronization criterion that combines the nonlinear property of the memristor and the framework of Lyapunov-Krasovskii functional (LKF) is proposed. Simulation results indicate that the proposed two approaches are useful for coupled MNNs, and they have less control cost for different types of quasi-synchronization.

## 1. Introduction

Recently, memristive neural networks (MNNs) have attracted much attention because of their widespread use in various fields, such as signal processing, image protection, and robotics (Hong et al., 2020; Yuan et al., 2020). The dynamical behaviors of the MNNs, such as chaos, synchronization, and stability, play a significant role in the above-mentioned applications (Duan et al., 2020; Liu H. et al., 2020). Synchronization is one of the most fundamental dynamic behaviors of MNNs, i.e., the approaching process of all interconnected nodes with different initial states in the systems. Therefore, based on the concept of traditional chaotic behavior, by using the synchronization of the drive-respond MNNs for multi-robot systems, the cooperative control issues of such systems can be redefined as the synchronization or consensus control issues of multi-agent systems (Wang C. et al., 2021). Accordingly, investigating the cooperative control algorithms based on the synchronization of MNNs is important, and numerous synchronization methods have been established to support robotic systems (Duong et al., 2022; Zhang Y. et al., 2022). Therefore, the synchronization of MNNs has innovative significance and prospects for multi-robot systems.

As a universal model of MNNs, coupled MNNs can imitate the human brain more truly than traditional MNNs. Considering this, the synchronization of coupled MNNs should be investigated and applied to the field of secure communication combined with the memristor that can mimic human synapses (Wang et al., 2019; Chen et al., 2021). A plethora of studies have been conducted to improve coupled MNNs for potential applications in image protection (Yuan et al., 2020), social networks (Zhu et al., 2022), pattern recognition, etc. Coupled neural networks have a coupled structure. By using the initial value sensitivity of the memristor, such networks can be applied to the field of image encryption with larger key space and higher security. However, when binary digital is extended to M-nary digital, the problem of faster communication still exists (Chee and Xu, 2006). To address this issue, projective synchronization was first introduced by Mainieri and Rehacek (1999).

In practice, different synchronizations are necessary between drive-response MNNs, especially for secure communication. Regarding the impact of the various projective factors (Chen et al., 2019; Fu et al., 2020; Ding et al., 2022) on different structures of neural networks, many research results have been obtained. Fu et al. (2020) studied the projective synchronization for fuzzy MNNs under a pinning control scheme. The fix-time projective synchronization with discrete-time delay was investigated by Chen et al. (2019). Considering the lag factor and the fractional structure of neural networks, the lag projective synchronization was studied by Ding et al. (2022). However, parameter mismatch is unavoidable in the synchronization mechanism. For time-varying delayed neural networks with parameter mismatch, projective synchronization was studied by Kumar et al. (2019). In Guo et al. (2020), the parameter-mismatch complex-valued neural networks realize quasi-projective synchronization with a linear feedback controller. Later, in Yang et al. (2021), the impulsive effect on weak projective synchronization was investigated for parameter-mismatch MNNs. However, the above studies did not consider uncertainty, and different models should be described to adapt to the complex situation for the practical requirements. Therefore, it is crucial to introduce time-varying delays and uncertainties in the modeling (Li et al., 2021; Rajchakit and Sriraman, 2021; Wu and Huang, 2022). This has inspired the authors to develop a less-conservative model to explore the synchronization of coupled MNNs.

Compared with complete synchronization, the error of synchronization should be allowed within a reasonable range for the robotic system, while keeping more operation resilience, and has been widely devoted to many practical applications, including underactuated robotic systems, underwater vehicles, and wheeled robots (Tang et al., 2016; Huang et al., 2018; Chen and Shan, 2019; Yao et al., 2020). Therefore, the investigation of quasi-synchronization is significant and necessary in theory and application. In Chen W. et al. (2022), the quasi-synchronization of a coupled neural network with fractional-order and time-varying delays was studied. Based on Halanay inequality and matrix measure techniques, quasi stability of inertial-delayed MNNs was discussed and implemented in Xin et al. (2022). In Jin et al. (2022), sufficient criteria of uncertain Lur'e networks were derived to guarantee the quasi-synchronization between dynamical systems. In Shi et al. (2022), considering the coupled heterogeneous harmonic oscillators, an event-triggered scheme was developed to ensure the quasi-bipartite synchronization under an undirected communication topology. In Zhang R. et al. (2022), under deception attacks, the time-space sampled-data control scheme was proposed to guarantee the quasi-synchronization for NNs having reaction-diffusion. However, from the perspective of engineering applications, most robotic systems are coupled systems with strong couplings among the master and slave states, therefore, the traditional control method in such a complex environment will lead to more energy consumption. Therefore, how to achieve a better quasi-synchronization effect in an energy-saving manner is still challenging.

Regarding this problem, an event-triggered scheme has been proposed to reduce computational costs under limited communication resources. For this scheme, the controller will not be updated until a certain triggered condition is satisfied. That is, the triggered function is the key to the event-triggered scheme. Apparently, a reasonable event-triggered scheme can realize synchronization and task execution with low energy consumption. In Zhu and Bao (2022), an event-triggered controller was introduced to explore the synchronization issue of coupled MNNs. In Cheng (2022), for multi-agent systems, an event-triggered method was designed to realize the output synchronization and the Zeno behavior was avoided effectively. In Li et al. (2022), two event-triggered impulsive control methods were proposed, and sufficient criteria were formulated to guarantee the globally exponential stability of impulsive systems. Till now, several effective event-triggering schemes for quasi-synchronization have been investigated by Zhou and Zeng (2019), Yan et al. (2020), and Hu et al. (2022). In Hu et al. (2022), for quasi-synchronization, an event-triggered communication mechanism under the switching topology was designed for complex NNs. Then, the hybrid event-triggered scheme was investigated to solve the quasi-synchronization problem for delayed MNNs with a novel threshold function (Zhou and Zeng, 2019; Yan et al., 2020). The above discussion indicates that the event-triggered scheme can ensure the controller to update if the variation of error exceeds the arbitrary threshold. Therefore, it is a challenging but significant issue to define event-triggered conditions including the projective factor, uncertainties, and parameter mismatch.

This article proposed an event-triggered scheme to achieve projective quasi-synchronization for coupled MNNs with time-varying delays and uncertainties. The novelties of this article include:

1. Different from general coupled MNNs, the time-varying uncertainties are considered by the proposed model, which is a type of uncertain switching system. Meanwhile, the definition of projective quasi-synchronization is first proposed based on such a system.
2. To effectively reduce energy consumption, the projective factor and time-varying uncertainties are introduced into the arbitrary triggered function of an event-triggered scheme to further extend the time span and decrease the control cost.
3. The projective quasi-synchronization criteria are formulated by designing a novel time-dependent and piecewise LKF. Meanwhile, different types of quasi-synchronization are illustrated, and an explicit error bound is provided. Besides, the Zeno behavior can be eliminated naturally.

For the rest of this article, Section 2 presents the model of coupled MNNs and the involved definitions, assumptions, as well as lemmas. Then, in Section 3, the theoretical analysis results are given, including a theorem and two corollaries. To validate the main results, two numerical examples are introduced in Section 4. Section 5 concludes the whole study.

## 2. Preliminaries of the neural networks model

### 2.1. Coupled MNNs model

Regard a class of delayed MNNs described as follows

(1) $$\begin{array}{l} \begin{array}{l} c_{l}\frac{\text{d}x_{l}\left(t\right)}{\text{d}t}=-\left[\left({𝕄}_{flm}+{\mathbb{N}}_{glm}\right)+\frac{1}{{ℜ}_{l}}\right]x_{l}\left(t\right)+\overset{n}{\underset{m=1}{\sum }}{𝕄}_{lm}\\ \times {\text{sgn}}_{lm}f_{m}\left(x_{m}\left(t\right)\right)+\overset{n}{\underset{m=1}{\sum }}{\mathbb{N}}_{flm}\times {\text{sgn}}_{lm}f_{m}\left(x_{m}\left(t-\tau \left(t\right)\right)\right)+I_{l}\left(t\right),\\ t\leq 0,l=1,2,\ldots ,n, \end{array} \end{array}$$

where *x*_*l*_(*t*) shows the voltage of the capacitor *c*_*l*_; the memristance of memristor between the feedback function *f*_*m*_(*x*_*m*_(*t*)) and *x*_*l*_(*t*) is illustrated as 𝕄_*flm*_; then, ℕ_*flm*_ denotes the memristance between the feedback function *f*_*m*_(*x*_*m*_(*t* − τ(*t*))) and *x*_*l*_(*t*). ℜ_*l*_ is the resistor, and *I*_*l*_(*t*) is an external bias or input. Besides, sgn_*lm*_ = 1 for *l* ≠ *m* and sgn_*lm*_ = −1 for *l* = *m*.

**Figure 1:**
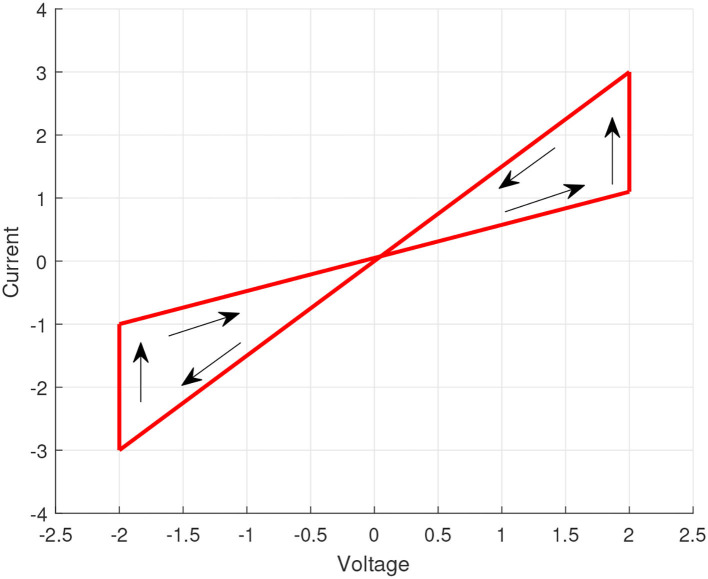
Typical current-voltage characteristic of a memristor.

Simplifying the mathematical model of the memristor is helpful to obtain the pinched hysteresis feature, so we select a surrogate model as shown in Figure 1.

Then, the state formula of MNNs with time-varying delays and uncertainties is shown in Equation (1).

(2) $$\begin{array}{l} \begin{array}{l} {\dot{x}}_{l}\left(t\right)=-d_{l}\left(x_{l}\left(t\right)\right)x_{l}\left(t\right)+\overset{n}{\underset{m=1}{\sum }}\left(a_{lm}\left(x_{l}\left(t\right)\right)+\Delta a_{lm}\left(t\right)\right)f_{m}\left(x_{m}\left(t\right)\right)\\ \;+\overset{n}{\underset{m=1}{\sum }}\left(b_{lm}\left(x_{l}\left(t\right)\right)+\Delta b_{lm}\left(t-\tau \left(t\right)\right)\right)f_{m}\left(x_{m}\left(t-\tau \left(t\right)\right)\right)+I_{l}\left(t\right), \end{array} \end{array}$$

where *f*_*m*_(*x*_*m*_(*t*)) and *f*_*m*_(*x*_*m*_(*t* − τ(*t*))) are the neural feedback functions; Δ*a*_*lm*_(*t*) and Δ*b*_*lm*_(*t* − τ(*t*)) are the time-varying uncertainties that satisfy |Δ*a*_*lm*_(*t*)| ≤ *a*_*lm*_ and |Δ*b*_*lm*_(*t* − τ(*t*))| ≤ *b*_*lm*_; *x*_*l*_(*t*) is the state of the *l* − *th* neuron, and τ(*t*) is the time-varying delay that meets 0 ≤ τ(*t*) ≤ τ. *d*_*l*_(*x*_*l*_(*t*)) is the *lth* neuron self-inhibition, *a*_*lm*_(*x*_*l*_(*t*)) and *b*_*lm*_(*x*_*l*_(*t* − τ(*t*))) represent the memristors synaptic connection weights memristor-based weights, and

(3) $$\begin{array}{l} \begin{array}{l} d_{l}\left(x_{l}\left(t\right)\right)=\frac{1}{c_{l}}\left[\overset{n}{\underset{m=1}{\sum }}\left({𝕄}_{flm}+{\mathbb{N}}_{flm}\right)+\frac{1}{{ℜ}_{l}}\right],\\ a_{lm}\left(x_{l}\left(t\right)\right)=\frac{{𝕄}_{flm}}{c_{l}}\times {\text{sgn}}_{lm},\\ b_{lm}\left(x_{l}\left(t-\tau \left(t\right)\right)\right)=\frac{{\mathbb{N}}_{flm}}{c_{l}}\times {\text{sgn}}_{lm}. \end{array} \end{array}$$

According to the characteristics of memristor, this paper designs the corresponding connection weights *d*_*l*_(*x*_*l*_(*t*)), *a*_*lm*_(*x*_*l*_(*t*)), and *b*_*lm*_(*x*_*l*_(*t*)) as follows

(4) $$\begin{array}{l} d_{l}\left(x_{l}\left(t\right)\right)=\{\begin{array}{r} {\hat{d}}_{l},\;x_{l}\left(t\right)>0,\\ \text{unchanged},\;x_{l}\left(t\right)=0,\\ {\overset{ˇ}{d}}_{l},\;x_{l}\left(t\right)<0, \end{array}\\ a_{lm}\left(x_{l}\left(t\right)\right)=\{\begin{array}{r} {â}_{lm},\;x_{l}\left(t\right)>0,\\ \text{unchanged},\;x_{l}\left(t\right)=0,\\ {ǎ}_{lm},\;x_{l}\left(t\right)<0, \end{array}\\ b_{lm}\left(x_{l}\left(t\right)\right)=\{\begin{array}{r} {\hat{b}}_{lm},\;x_{l}\left(t\right)>0,\\ \text{unchanged},\;x_{l}\left(t\right)=0,\\ {\overset{ˇ}{b}}_{lm},\;x_{l}\left(t\right)<0, \end{array} \end{array}$$

where *l, m* = 1, 2, …, *n*; ${\hat{d}}_{l}$, ${\overset{ˇ}{d}}_{l}$, â_*lm*_, ǎ_*lm*_, ${\hat{b}}_{lm}$, and ${\overset{ˇ}{b}}_{lm}$ are constants. The corresponding response system connection weights *d*_*l*_(*y*_*l*_(*t*)), *a*_*lm*_(*y*_*l*_(*t*)), and *b*_*lm*_(*y*_*l*_(*t*)) are defined in a similar way.

On the basis of the solution to such a system by Filippov, let

(5) $$\begin{array}{l} \begin{array}{l} \;{\bar{d}}_{l}=\text{max}\left\{\left|{\hat{d}}_{l}|,|{\overset{ˇ}{d}}_{l}\right|\right\},{\underline{d}}_{l}=\text{min}\left\{\left|{\hat{d}}_{l}|,|{\overset{ˇ}{d}}_{l}\right|\right\},\\ {ā}_{lm}=\text{max}\left\{\left|{â}_{lm}|,|{ǎ}_{lm}\right|\right\},{\underline{a}}_{lm}=\text{min}\left\{\left|{â}_{lm}|,|{ǎ}_{lm}\right|\right\},\\ {\bar{b}}_{lm}=\text{max}\left\{\left|{\hat{b}}_{lm}|,|{\hat{b}}_{lm}\right|\right\},{\underline{b}}_{lm}=\text{min}\left\{\left|{\hat{b}}_{lm}|,|{\overset{ˇ}{b}}_{lm}\right|\right\}, \end{array} \end{array}$$

and there exist measurable functions ${\tilde{d}}_{l}\left(x_{l}\left(t\right)\right)\in \left[{\bar{d}}_{l},{\underline{d}}_{l}\right]$, ${\tilde{a}}_{lm}\left(x_{l}\left(t\right)\right)\in \left[{ā}_{lm},{\underline{a}}_{lm}\right]$, ${\tilde{b}}_{lm}\left(x_{l}\left(t\right)\right)\in \left[{\bar{b}}_{lm},{\underline{b}}_{lm}\right]$.

Combined with the differential inclusion theory and the set-valued map theory, the drive system (1) with an initial value $x\left(s\right)=\phi \left(s\right)={\left(\phi_{1}\left(s\right),\phi_{2}\left(s\right),\ldots ,\phi_{n}\left(s\right)\right)}^{\text{T}}\in C\left(\left[-\tau ,0\right],{\mathbb{R}}^{n}\right)$ is described as follows:

(6) $$\begin{array}{l} \begin{array}{l} {\dot{x}}_{l}\left(t\right)=-{\tilde{d}}_{l}\left(x_{l}\left(t\right)\right)x_{l}\left(t\right)+\overset{n}{\underset{m=1}{\sum }}\left({\tilde{a}}_{lm}\left(x_{l}\left(t\right)\right)+\Delta a_{lm}\left(t\right)\right)f_{m}\left(x_{m}\left(t\right)\right)\\ \;+\overset{n}{\underset{m=1}{\sum }}\left({\tilde{b}}_{lm}\left(x_{l}\left(t\right)\right)+\Delta b_{lm}\left(t-\tau \left(t\right)\right)\right)f_{m}\left(x_{m}\left(t-\tau \left(t\right)\right)\right)\\ \;+I_{l}\left(t\right). \end{array} \end{array}$$

The response system in the initial conditions $y\left(s\right)=\varphi \left(s\right)={\left(\varphi_{1}\left(s\right),\varphi_{2}\left(s\right),\ldots ,\varphi_{n}\left(s\right)\right)}^{\text{T}}\in C\left(\left[-\tau ,0\right],{\mathbb{R}}^{n}\right)$ is

(7) $$\begin{array}{l} \begin{array}{l} {ẏ}_{l}\left(t\right)=-{\tilde{d}}_{l}\left(y_{l}\left(t\right)\right)y_{l}\left(t\right)+\overset{n}{\underset{m=1}{\sum }}\left({\tilde{a}}_{lm}\left(y_{l}\left(t\right)\right)+\Delta a_{lm}^{*}\left(t\right)\right)f_{m}\left(y_{m}\left(t\right)\right)\\ \;+\overset{n}{\underset{m=1}{\sum }}\left({\tilde{b}}_{lm}\left(y_{l}\left(t\right)\right)+\Delta b_{lm}^{*}\left(t-\tau \left(t\right)\right)\right)f_{m}\left(y_{m}\left(t-\tau \left(t\right)\right)\right)\\ \;+U_{l}\left(t\right)+I_{l}\left(t\right), \end{array} \end{array}$$

where $\Delta a_{lm}^{*}\left(t\right)$ and $\Delta b_{lm}^{*}\left(t-\tau \left(t\right)\right)$ are time-varying uncertainties that satisfy $|\Delta a_{lm}^{*}\left(t\right)|\leq a_{lm}^{*}$ and $|\Delta b_{lm}^{*}\left(t-\tau \left(t\right)\right)|\leq b_{lm}^{*}$; *U*_*l*_(*t*) represents the appropriate control input to be designed.

The coupled MNNs containing *N* identical MNNs are represented as follows

(8) $$\begin{array}{l} \begin{array}{l} \dot{x}\left(t\right)=-\tilde{D}\left(x\left(t\right)\right)x\left(t\right)+\left(\tilde{A}\left(x\left(t\right)\right)+\Delta A\left(t\right)\right)f\left(x\left(t\right)\right)\\ \;+\left(\tilde{B}\left(x\left(t\right)\right)+\Delta B\left(t-\tau \left(t\right)\right)\right)f\left(x\left(t-\tau \left(t\right)\right)\right)+\sigma \Gamma Wx\left(t\right)+I\left(t\right), \end{array} \end{array}$$

and

(9) $$\begin{array}{l} \begin{array}{l} ẏ\left(t\right)=-\tilde{D}\left(y\left(t\right)\right)y\left(t\right)+\left(\tilde{A}\left(y\left(t\right)\right)+\Delta A^{*}\left(t\right)\right)f\left(y\left(t\right)\right)\\ \;+(\tilde{B}\left(y\left(t\right)\right)+\Delta B^{*}\left(\left(t-\tau \left(t\right)\right)\right)f\left(y\left(t-\tau \left(t\right)\right)\right)+\sigma \Gamma Wy\left(t\right)\\ \;+U\left(t\right)+I\left(t\right), \end{array} \end{array}$$

where $x\left(t\right)={\left(x_{1}\left(t\right),x_{2}\left(t\right),\ldots ,x_{N}\left(t\right)\right)}^{\text{T}}$ and $y\left(t\right)={\left(y_{1}\left(t\right),y_{2}\left(t\right),\ldots ,y_{N}\left(t\right)\right)}^{\text{T}}$ are the states of the drive and response system. The response activation functions are $f\left(x\left(t\right)\right)={\left(f_{1}\left(x_{1}\left(t\right)\right),f_{2}\left(x_{1}\left(t\right)\right),\ldots ,f_{N}\left(x_{N}\left(t\right)\right)\right)}^{\text{T}}$, and $f\left(y\left(t\right)\right)={\left(f_{1}\left(y_{1}\left(t\right)\right),f_{2}\left(y_{1}\left(t\right)\right),\ldots ,f_{N}\left(y_{N}\left(t\right)\right)\right)}^{\text{T}}$. For the structure of the coupled system, this article defines a coupled matrix *W* = (*w*_*ij*_)_*N*×*N*_, *i, j* = 1, 2, …, *N*, which satisfies *H*1: If there is a direct edge from *j* to *i*, then *w*_*ij*_ = 1; otherwise, *w*_*ij*_ = 0; *H*2: For *i, j* = 1, 2, …, *N*, the diffusive coupling conditions are satisfied as $w_{ij}=-\overset{N}{\underset{j=1,j\neq i}{\sum }}w_{ij}$. σ > 0 denotes the coupling strength, and Γ refers to the inner couple matrix.

The projective synchronization error is defined as $\varepsilon \left(t\right)={\left(y_{1}\left(t\right)-\alpha x_{1}\left(t\right),y_{2}\left(t\right)-\alpha x_{2}\left(t\right),\ldots ,y_{N}\left(t\right)-\alpha x_{N}\left(t\right)\right)}^{\text{T}}={\left(\varepsilon_{1}\left(t\right),\varepsilon_{2}\left(t\right),\ldots ,\varepsilon_{N}\left(t\right)\right)}^{\text{T}}\in C(\left[-\tau ,0\right],{\mathbb{R}}^{n}$, where α represents the projective factor.

Then, the synchronization issue can be regarded as the stability of the error system:

(10) $$\begin{array}{l} \begin{array}{l} \dot{\varepsilon }\left(t\right)=y\left(t\right)-\alpha x\left(t\right)\\ \;=-\tilde{D}\left(y\left(t\right)\right)y\left(t\right)+\alpha \tilde{D}\left(x\left(t\right)\right)x\left(t\right)+\left(\tilde{A}\left(y\left(t\right)\right)+\Delta A^{*}\left(t\right)\right)f\left(y\left(t\right)\right)\\ \;-\alpha \left(\tilde{A}\left(x\left(t\right)\right)+\Delta A\left(t\right)\right)f\left(x\left(t\right)\right)+\sigma \Gamma Wy\left(t\right)-\alpha \sigma \Gamma Wx\left(t\right)\\ \;+(\tilde{B}\left(y\left(t\right)\right)+\Delta B^{*}(\left(t-\tau \left(t\right)\right))f\left(y\left(t-\tau \left(t\right)\right)\right)\\ \;-\alpha \left(\tilde{B}\left(x\left(t\right)\right)+\Delta B\left(t-\tau \left(t\right)\right)\right)f\left(x\left(t-\tau \left(t\right)\right)\right)+U\left(t\right)\\ \;=-\tilde{D}\left(y\left(t\right)\right)\varepsilon \left(t\right)+\left(\tilde{A}\left(y\left(t\right)\right)+\Delta A^{*}\left(t\right)\right)f\left(\varepsilon \left(t\right)\right)+\alpha \sigma \Gamma W\varepsilon \left(t\right)\\ \;+(\tilde{B}\left(y\left(t\right)\right)+\Delta B^{*}\left(\left(t-\tau \left(t\right)\right)\right)f\left(\varepsilon \left(t-\tau \left(t\right)\right)\right)+U\left(t\right)+\text{Ψ}\left(t\right), \end{array} \end{array}$$

where $\text{Ψ}\left(t\right)=\left(\tilde{A}\left(y\left(t\right)\right)+\Delta A^{*}\left(t\right)\right)f\left(\alpha x\left(t\right)\right)-\alpha \left(\tilde{A}\left(x\left(t\right)\right)+\Delta A\left(t\right)\right)f\left(x\left(t\right)\right)+(\tilde{B}\left(y\left(t\right)\right)+\Delta B^{*}\left(\left(t-\tau \left(t\right)\right)\right)f\left(\alpha x\left(t-\tau \left(t\right)\right)\right)-\alpha \left(\tilde{B}\left(x\left(t\right)\right)+\Delta B\left(t-\tau \left(t\right)\right)\right)f\left(x\left(t-\tau \left(t\right)\right)\right)$, *f*(ε(*t*)) = *f*(*y*(*t*)) − *f*(α*x*(*t*)).

Then, the controller *U*(*t*) is designed as follows

(11) $$\begin{array}{l} \begin{array}{l} U\left(t\right)=K\varepsilon \left(t_{k-1}\right)+\overset{\infty }{\underset{k=1}{\sum }}\left(\mu_{k}-1\right)\varepsilon \left(t\right)\delta \left(t-t_{k}\right),k\in N_{+},\mu_{k}\neq 0, \end{array} \end{array}$$

where $K={\left(K_{1},K_{2},\ldots ,K_{N}\right)}^{\text{T}}$ represents the gain of state-feedback controller, $\mu_{k}\in R$ represents the impulse strength, and δ(*t*) represents the Dirac function. This article defines the initial time as *t*_0_ = 0, and the subsequent events determine the sequence of impulsive instants {*t*_1_, *t*_2_, *t*_3_, … }.

**Remark 1**. Enlighten by Zhou and Zeng (2019), we take the time-varying uncertainties and delays of information transmission into account of designing the triggered function. As a result, it is more accurate to describe the various influences from the external environment on the networks by using the variable of the uncertainties. On the other hand, the quasi-synchronization scheme for MMNs was presented by Zhou and Zeng (2019). However, in order to meet the practical application, more types of synchronization are considered. Therefore, the event-triggered rules in Yang et al. (2018), Liu et al. (2019), Liu J. et al. (2020), and Wang W. et al. (2021) can be derived from the newly developed event-triggered scheme in (11). Consequently, the proposed scheme is more universal and can be applied to more complex communication environments than other methods.

Assume that ε(*t*) is right continuous at *t* = *t*_*k*_, i.e., $\varepsilon \left(t_{k}\right)=\varepsilon \left(t_{k}^{+}\right)$. Therefore, the solution to the error system (10) is jumping discontinuously at *t* = *t*_*k*_, which indicates that the error system can change the state variables at *t* = *t*_*k*_ with the control method (11). Thus, the system (10) under a hybrid event-triggered impulse can be represented as:


(12) $$\begin{array}{l} \{\begin{array}{l} \;\dot{\varepsilon }\left(t\right)=-\tilde{D}\left(y\left(t\right)\right)\varepsilon \left(t\right)+\left(\tilde{A}\left(y\left(t\right)\right)+\Delta A^{*}\left(t\right)\right)f\left(\varepsilon \left(t\right)\right)+\alpha \sigma \Gamma W\varepsilon \left(t\right)\\ \;+(\tilde{B}\left(y\left(t\right)\right)+\Delta B^{*}\left(\left(t-\tau \left(t\right)\right)\right)f\left(\varepsilon \left(t-\tau \left(t\right)\right)\right)\\ \;+\text{Ψ}\left(t\right)+K\varepsilon \left(t_{k-1}\right),t\in \left[t_{k-1},t_{k}\right),k\in N_{+},\\ \varepsilon \left(t_{k}^{+}\right)=\mu_{k}t_{k}^{-},\mu_{k}\neq 0, \end{array} \end{array}$$

where *t* ≥ 0. The impulse control scheme only works at *t* = *t*_*k*_. Then, the measurement error of the error system (12) can be expressed as

(13) $$\begin{array}{l} \begin{array}{l} e\left(t\right)=\varepsilon \left(t_{k-1}\right)-\varepsilon \left(t\right),t\in \left[t_{k-1},t_{k}\right)k\in N_{+}, \end{array} \end{array}$$

where $e\left(t\right)={\left(e_{1}\left(t\right),e_{2}\left(t\right),\ldots ,e_{N}\left(t\right)\right)}^{\text{T}}$.

**Figure 2:**
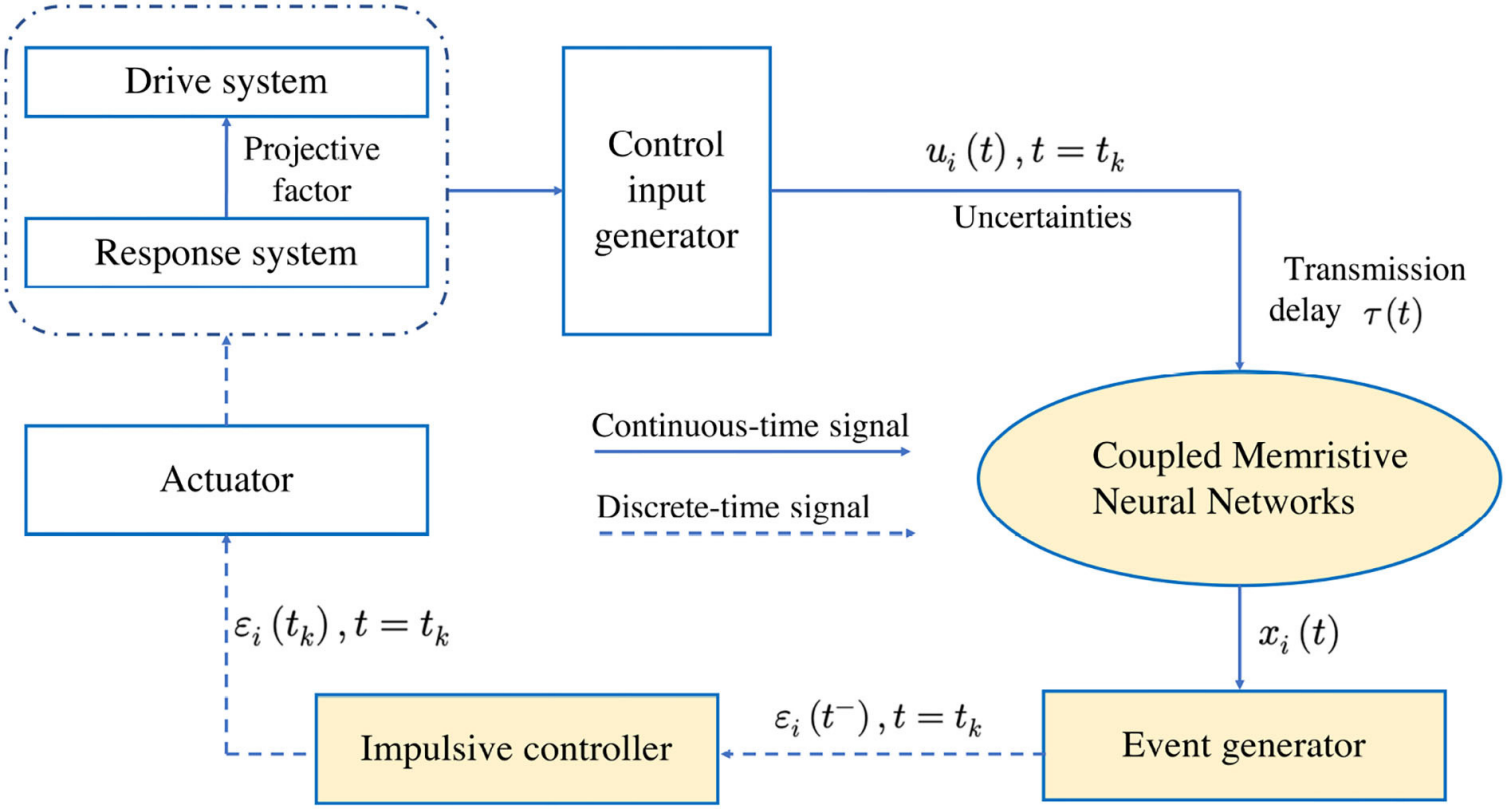
The event-triggered scheme for the proposed couple MNNs.

**Remark 2**. The event-triggered scheme for the proposed couple MNNs is illustrated in Figure 2 Comparing the currently available results, the projective factor for quasi-synchronization and uncertainties are taken into account for designing the triggered function, which determines the triggered instant *t*_*k*_. At each instant *t*_*k*_, the information ε_*i*_(*t*_*k*_) exchange is realized from the sensors to the event generators and actuators. At the same time, the designed event-triggered scheme is activated and the sampled has occurred in the event-generators. Then the control input *u*_*i*_(*t*_*k*_) works for coupled MNNs and the system enters the new loop according to the updated events.

Then, some definitions, assumptions, as well as lemmas are presented to investigate projective quasi-synchronization for systems (8) and (9).

### 2.2. Some useful definitions and assumptions

**Assumption 1**. (Fu et al., 2020) Assume that the solution to (8) is bounded for any initial values $x\left(s\right)=\phi \left(s\right)={\left(\phi_{1}\left(s\right),\phi_{2}\left(s\right),\ldots ,\phi_{n}\left(s\right)\right)}^{\text{T}}\in C\left(\left[-\tau ,0\right],{\mathbb{R}}^{n}\right)$, there is a positive constant *M* such that |*x*_*i*_(*t*)| ≤ *M* for all *t* ∈ [−τ, ∞).

**Assumption 2**. (Zhou and Zeng, 2019) The activation function *f*_*m*_(·) is Lipschitez continuous, and there exists a constant *f*_*m*_ > 0 such that

(14) $$\begin{array}{l} |f_{m}\left(s_{1}\right)-f_{m}\left(s_{2}\right)|\leq f_{m}|s_{1}-s_{2}|, \end{array}$$

for all *s*_1_, *s*_2_ ∈ ℝ, and *f*_*m*_(0) = 0.

**Definition 1**. (Tang et al., 2018) Systems (8) and (9) achieve projective quasi-synchronization with an error bound ϵ > 0 if the error ε(*t*) = *y*(*t*) − α*x*(*t*) exponentially converges into a compact set $𝔻:=\psi \left(s\right)=\left\{{\left(\varepsilon_{1}\left(s\right),\varepsilon_{2}\left(s\right),\ldots ,\varepsilon_{N}\left(s\right)\right)}^{\text{T}}\in C\left(\left[-\tau ,0\right],{\mathbb{R}}^{N}\right)|\overset{N}{\underset{i=1}{\sum }}\varepsilon_{i}^{2}\left(t\right)\leq \epsilon \right\}$ as *t* → ∞, where *x*(*t*) represents the solution to system (8) that has an initial value of ϕ(*s*), and *y*(*t*) represents the solution to system (9) that has an initial value of φ(*s*).

**Definition 2**. (Zhu et al., 2017) The error system (10) can eliminate Zeno behaviors if a constant ρ > 0 exists such that

(15) $$\begin{array}{l} \underset{k\in N_{+}}{\text{inf}}\left\{t_{k}-t_{k-1}\right\}\geq \rho >0. \end{array}$$

**Lemma 1**. Assuming that the real matrices *P* and *Q* have appropriate dimensions, a positive number α exists such that

(16) $$\begin{array}{l} P^{\text{T}}Q+Q^{\text{T}}P\leq \alpha P^{\text{T}}Q+\alpha^{-1}Q^{\text{T}}P \end{array}$$

**Lemma 2**. (Zhang et al., 2009) Let ν_1_(*t*) and ν_2_(*t*) ∈ ℙℂ be jumping discontinuously at *t* = *t*_*k*_, *t* ≥ 0, for $\nu_{1}\left(t_{k}^{-}\right)$ and $\nu_{2}\left(t_{k}^{-}\right)$, there is $\nu_{1}\left(t_{k}^{-}\right)=\nu_{1}\left(t_{k}^{+}\right)$ and $\nu_{2}\left(t_{k}^{-}\right)=\nu_{2}\left(t_{k}^{+}\right)$. If there are constants β_1_ > 0, β_2_ > 0, and μ > 0 such that

(17) $$\begin{array}{l} \{\begin{array}{l} {\text{D}}^{+}\nu_{1}\left(t\right)\leq \beta_{1}\nu_{1}\left(t\right)+\beta_{2}\nu_{1}\left(t-\tau \left(t\right)\right),t\neq t_{k},t\geq 0\\ \;\nu_{1}\left(t_{k}^{+}\right)\leq \mu \nu_{1}\left(t_{k}^{-}\right),k\in N_{+}, \end{array} \end{array}$$

and

(18) $$\begin{array}{l} \{\begin{array}{l} {\text{D}}^{+}\nu_{2}\left(t\right)>\beta_{1}\nu_{2}\left(t\right)+\beta_{2}\nu_{2}\left(t-\tau \left(t\right)\right),t\neq t_{k},t\geq 0\\ \;\nu_{2}\left(t_{k}^{+}\right)\leq \mu \nu_{2}\left(t_{k}^{-}\right),k\in N_{+}, \end{array} \end{array}$$

and ν_1_(*t*) ≤ ν_2_(*t*) for −τ ≤ *t* ≤ 0, then ν_1_(*t*) ≤ ν_2_(*t*) for *t* > 0.

## 3. Main results

Here, this article presents the solution to the exponential projective quasi-synchronization of delayed uncertain coupled MNNs under the proposed event-triggered strategy.

For convenience, we make *A* = (*a*_*lm*_)_*n*×*n*_, $A^{*}={\left(a_{lm}^{*}\right)}_{n\times n}$, *B* = (*b*_*lm*_)_*n*×*n*_, $B^{*}={\left(b_{lm}^{*}\right)}_{n\times n}$, *F* = diag{*f*_1_, *f*_2_, …, *f*_*n*_}, $\bar{D}=\text{diag}\left\{{\bar{d}}_{1},{\bar{d}}_{2},...,{\bar{d}}_{n}\right\}$, *D* = diag{*d*_1_, *d*_2_, …, *d*_*n*_}, Ā = (ā_*lm*_)_*n*×*n*_, *A* = (*a*_*lm*_)_*n*×*n*_, $\bar{B}={\left({\bar{b}}_{lm}\right)}_{n\times n}$, *B* = (*b*_*lm*_)_*n*×*n*_, $\Gamma_{3}=\bar{B}+B^{*}$.

**Theorem 1**. *According to Assumptions 1 and 2, systems (8) and (9) achieve exponentially projective quasi-synchronization with the control law (11), if there are constants ρ*_1_ > 0, *α*_*i*_ > 0 (*i* = 1, 2, 3), $\tilde{\alpha }>0$, *ω* > 0 *such that*

(19) $$\begin{array}{l} \mu_{k}^{2}\geq 1, \end{array}$$

(20) $$\begin{array}{l} \Lambda <0, \end{array}$$

(21) $$\begin{array}{l} -\left(\frac{ln\tilde{\mu }}{\rho_{1}}-\omega \right)>\tilde{\mu }\beta >0, \end{array}$$

(22) $$\begin{array}{l} \tilde{\alpha }-\frac{1}{2}\alpha^{2}\alpha_{1}-\frac{1}{2}\alpha_{2}\Gamma_{3}^{T}\Gamma_{3}-\frac{1}{2}\alpha K^{2}>0, \end{array}$$

(23) $$\begin{array}{l} \eta \left(t\right)<0,\;t\in \left[t_{k-1},t_{k}\right) \end{array}$$

where *k* ∈ *N*_+_, $\Lambda =\tilde{\alpha }+\omega -\lambda_{max}\left(Ā-\bar{D}+\bar{B}A^{*}+B^{*}+\sigma \Gamma W+K\right)$, $\beta =\frac{1}{2}\alpha_{2}^{-1}F^{2}$, $\tilde{\mu }=\underset{k}{max}\left\{\mu_{k}^{2}\right\}$.

In this article, the triggered function is designed as

(24) $$\begin{array}{l} \begin{array}{l} \eta \left(t\right)=||e\left(t\right)|{|}^{2}-2\alpha_{2}|\varepsilon \left(t\right)|\left(\tilde{\alpha }-\frac{1}{2}\alpha^{2}\alpha_{1}-\frac{1}{2}\alpha_{2}\Gamma_{3}^{\text{T}}\Gamma_{3}-\frac{1}{2}\alpha K^{2}\right)\\ \;|\varepsilon \left(t\right)| \end{array} \end{array}$$

The triggered instant *t*_*k*_ depends on the following event-triggered condition

(25) $$\begin{array}{l} t_{k}=\underset{t}{\text{inf}}\left\{t\in \left(t_{k-1},\infty \right)|\eta \left(t\right)\geq 0\right\}. \end{array}$$

*Proof:* For system (12), the nonnegative Lyapunov function is adopted

(26) $$\begin{array}{l} V\left(t\right)=\frac{1}{2}\varepsilon^{\text{T}}\left(t\right)\varepsilon \left(t\right). \end{array}$$

By differentiating *V*(*t*) and the solution to (12) for the [*t*_*k*−1_, *t*_*k*_),*k* ∈ *N*_+_, there is

(27) $$\begin{array}{l} {\text{D}}^{+}V\left(t\right)=\varepsilon^{\text{T}}\left(t\right)\dot{\varepsilon }\left(t\right). \end{array}$$

Combining (12) and (27), there is

(28) $$\begin{array}{l} \begin{array}{l} {\text{D}}^{+}V\left(t\right)=\varepsilon^{\text{T}}\left(t\right)[-\tilde{D}\left(y\left(t\right)\right)\varepsilon \left(t\right)+\left(\tilde{A}\left(y\left(t\right)\right)+\Delta A^{*}\left(t\right)\right)f\left(\varepsilon \left(t\right)\right)\\ \;+(\tilde{B}\left(y\left(t\right)\right)+\Delta B^{*}\left(\left(t-\tau \left(t\right)\right)\right)f\left(\varepsilon \left(t-\tau \left(t\right)\right)\right)+\alpha \sigma \Gamma W\varepsilon \left(t\right)\\ \;+\text{Ψ}\left(t\right)+K\varepsilon \left(t_{k-1}\right)] \end{array} \end{array}$$

Consider Assumption 2, we have

(29) $$\begin{array}{l} f\left(\varepsilon \left(t\right)\right)-f\left(0\right)\leq F\varepsilon \left(t\right). \end{array}$$

Then, taking the parameters mismatched rules and uncertainties of systems (8) and (9), we have

(30) $$\begin{array}{l} \begin{array}{l} {\text{D}}^{+}V\left(t\right)\leq |\varepsilon^{\text{T}}\left(t\right)|\underline{D}|\varepsilon \left(t\right)|+|\varepsilon^{\text{T}}\left(t\right)|\left(Ā+A^{*}\right)F|\varepsilon \left(t\right)|+|\varepsilon^{\text{T}}\left(t\right)|\sigma \\ \;\Gamma W|\varepsilon \left(t\right)|+|\varepsilon^{\text{T}}\left(t\right)|\left(\bar{B}+B^{*}\right)F|\varepsilon \left(t-\tau \left(t\right)\right)|+|\varepsilon^{\text{T}}\left(t\right)|\text{Ψ}\left(t\right)\\ \;+|\varepsilon^{\text{T}}\left(t\right)|K|\varepsilon \left(t\right)+e\left(t\right)|. \end{array} \end{array}$$

For Ψ(*t*), by Assumptions 1–2 and Lemma 1, there are

(31) $$\begin{array}{l} \begin{array}{l} \Delta B^{*}\left(t\right)f\left(\alpha x\left(t-\tau \left(t\right)\right)\right)-\alpha \Delta B\left(t-\tau \left(t\right)\right)f\left(x\left(t-\tau \left(t\right)\right)\right)\leq \alpha \left(B^{*}-B\right)M.\\ \tilde{D}\left(x\left(t\right)\right)\alpha x\left(t\right)-\tilde{D}\left(y\left(t\right)\right)\alpha x\left(t\right)\leq \alpha \left(\bar{D}-\underline{D}\right)M,\\ \tilde{A}\left(y\left(t\right)\right)f\left(\alpha x\left(t\right)\right)-\alpha \tilde{A}\left(x\left(t\right)\right)f\left(x\left(t\right)\right)\leq \alpha \left(Ā-\underline{A}\right)M,\\ \tilde{B}\left(y\left(t\right)\right)f\left(\alpha x\left(t-\tau \left(t\right)\right)\right)-\alpha \tilde{B}\left(x\left(t\right)\right)f\left(x\left(t-\tau \left(t\right)\right)\right)\leq \alpha \left(Ā-\underline{B}\right)M,\\ \Delta A^{*}\left(t\right)f\left(\alpha x\left(t\right)\right)-\alpha \Delta A\left(t\right)f\left(x\left(t\right)\right)\leq \alpha \left(A^{*}-A\right)M. \end{array} \end{array}$$

As mentioned above, there is

(32) $$\begin{array}{l} \text{Ψ}\left(t\right)\leq \Gamma_{2}\alpha M, \end{array}$$

where $\Gamma_{2}=\bar{D}+Ā+\bar{B}+A^{*}+B^{*}-\underline{D}-\underline{A}-\underline{B}-A-B$.

Then, considering Lemma 1, there is

(33) $$\begin{array}{l} \begin{array}{l} |\varepsilon^{\text{T}}\left(t\right)|\text{Ψ}\left(t\right)\leq |\varepsilon^{\text{T}}\left(t\right)|\Gamma_{2}\alpha M\\ \;\leq \frac{1}{2}\alpha^{2}\alpha_{1}|\varepsilon^{\text{T}}\left(t\right)||\varepsilon \left(t\right)|+\frac{1}{2}\alpha_{1}^{-1}M^{\text{T}}\Gamma_{2}^{\text{T}}\Gamma_{2}M. \end{array} \end{array}$$

Let $\Gamma_{3}=\bar{B}+B^{*}$, then there is

(34) $$\begin{array}{l} \begin{array}{l} \left|\varepsilon^{\text{T}}\left(t\right)|\Gamma_{3}F|\varepsilon \left(t-\tau \left(t\right)\right)\right|\\ \leq \frac{1}{2}\alpha_{2}|\varepsilon^{\text{T}}\left(t\right)|\Gamma_{3}^{\text{T}}\Gamma_{3}|\varepsilon \left(t\right)|+\frac{1}{2}\alpha_{2}^{-1}|\varepsilon^{\text{T}}\left(t-\tau \left(t\right)\right)|F^{2}|\varepsilon \left(t-\tau \left(t\right)\right)|, \end{array} \end{array}$$

and

(35) $$\begin{array}{l} \begin{array}{l} \left|\varepsilon^{\text{T}}\left(t\right)|K|\varepsilon \left(t\right)+e\left(t\right)\right|\\ =|\varepsilon^{\text{T}}\left(t\right)|K|\varepsilon \left(t\right)|+\frac{1}{2}\alpha_{3}|\varepsilon^{\text{T}}\left(t\right)|K^{2}|\varepsilon \left(t\right)|+\frac{1}{2}\alpha_{3}^{-1}|e^{\text{T}}\left(t\right)||e\left(t\right)|\\ =|\varepsilon^{\text{T}}\left(t\right)|\left(K+\frac{1}{2}\alpha_{3}K^{2}\right)|\varepsilon \left(t\right)|+\frac{1}{2}\alpha_{3}^{-1}|e^{\text{T}}\left(t\right)||e\left(t\right)|. \end{array} \end{array}$$

Substituting (28)–(35) into (27) yields

(36) $$\begin{array}{l} \begin{array}{l} {\text{D}}^{+}V\left(t\right)\leq |\varepsilon^{\text{T}}\left(t\right)|[-\underline{D}+Ā+\bar{B}+A^{*}+B^{*}+\sigma \Gamma W\\ \;+\frac{1}{2}\alpha^{2}\alpha_{1}+\frac{1}{2}\alpha_{2}\Gamma_{3}^{\text{T}}\Gamma_{3}+K+\frac{1}{2}\alpha_{3}K^{2}]|\varepsilon \left(t\right)|\\ \;+\frac{1}{2}\alpha_{2}^{-1}|\varepsilon^{\text{T}}\left(t-\tau \left(t\right)\right)|F^{2}|\varepsilon \left(t-\tau \left(t\right)\right)|\\ \;+\frac{1}{2}\alpha_{3}^{-1}|e^{\text{T}}\left(t\right)||e\left(t\right)|+\frac{1}{2}\alpha_{1}^{-1}M^{\text{T}}\Gamma_{2}^{\text{T}}\Gamma_{2}M. \end{array} \end{array}$$

According to (22) and (23), there is

(37) $$\begin{array}{l} \begin{array}{l} {\text{D}}^{+}V\left(t\right)\leq |\varepsilon^{\text{T}}\left(t\right)|\left[-\underline{D}+Ā+\bar{B}+A^{*}+B^{*}+\sigma \Gamma W+K+\tilde{\alpha }\right]\\ \;|\varepsilon \left(t\right)|+\frac{1}{2}\alpha_{2}^{-1}|\varepsilon^{\text{T}}\left(t-\tau \left(t\right)\right)|F^{2}|\varepsilon \left(t-\tau \left(t\right)\right)|+\kappa , \end{array} \end{array}$$

where $\kappa =\frac{1}{2}\alpha_{1}^{-1}M^{\text{T}}\Gamma_{2}^{\text{T}}\Gamma_{2}M$.

From Lemma 2, the following observation can be obtained

(38) $$\begin{array}{l} \begin{array}{l} {\text{D}}^{+}V\left(t\right)\leq -\omega V\left(t\right)+\beta V\left(t-\tau \left(t\right)\right)+\kappa . \end{array} \end{array}$$

When *t* = *t*_*k*_, *k* ∈ *N*_+_, consider (27),

(39) $$\begin{array}{l} \begin{array}{l} V\left(t_{k}^{+}\right)={\left(\mu_{k}\varepsilon \left(t_{k}^{-}\right)\right)}^{\text{T}}\left(\mu_{k}\varepsilon \left(t_{k}^{-}\right)\right)=\mu_{k}^{2}V\left(t_{k}^{-}\right). \end{array} \end{array}$$

When δ > 0, ν(*t*) is developed as a unique solution to the proposed coupled MNNs, i.e.,

(40) $$\begin{array}{l} \{\begin{array}{l} D^{+}\nu \left(t\right)=-\omega \nu \left(t\right)+\beta \nu \left(t-\tau \left(t\right)\right)+\delta +\kappa ,t\neq t_{k},t\geq 0,\\ \;\nu \left(t_{k}^{+}\right)=\mu_{k}^{2}\nu \left(t_{k}^{-}\right),k\in N_{+},\\ \;\nu \left(t\right)=||\varepsilon \left(t\right)|{|}^{2},-\tau \leq t\leq 0. \end{array} \end{array}$$

According to Lemma 2,

(41) $$\begin{array}{l} V\left(t\right)\leq \nu \left(t\right),t\geq 0. \end{array}$$

Then,

(42) $$\begin{array}{l} \nu \left(t\right)=W\left(t\right)+\int_{0}^{t}W\left(t\right)\left(\beta_{1}\nu \left(s-\tau \left(s\right)\right)+\delta +\kappa \right)\text{d}s,t\geq 0, \end{array}$$

where *W*(*t*) represents the Cauchy matrix for Equations (17) and (18), i.e.,

(43) $$\begin{array}{l} \{\begin{array}{l} D^{+}\nu \left(t\right)=-\omega \nu \left(t\right),t\neq t_{k},t\geq 0.\\ \;\nu \left(t_{k}^{+}\right)=\mu_{k}^{2}\nu \left(t_{k}^{-}\right),k\in N_{+}, \end{array} \end{array}$$

then we have $W\left(t\right)={\text{e}}^{-\omega \left(t-s\right)}\underset{s\leq t_{k}\leq t}{\Pi }\mu_{k}^{2}$.

Considering Theorem 1, there is $\underset{k\in N_{+}}{\text{inf}}${*t*_*k*_ − *t*_*k*−1_} > 0, and a constant ρ_1_ satisfies $\underset{k\in N_{+}}{\text{inf}}${*t*_*k*_ − *t*_*k*−1_} ≥ ρ_1_ > 0. Then, we have

(44) $$\begin{array}{l} W\left(t\right)\leq {\text{e}}^{-\omega \left(t-s\right)}{\tilde{\mu }}^{\left(\frac{t-s}{\rho_{1}}+1\right)}\leq \tilde{\mu }{\text{e}}^{\left(\frac{\text{ln}\tilde{\mu }}{\rho_{1}}-\omega \right)\left(t-s\right)}, \end{array}$$

and $\tilde{\mu }=\underset{k}{\text{max}}\left\{\mu_{k}^{2}\right\}$.

Substituting (44) into (42) yields

(45) $$\begin{array}{l} \nu \left(t\right)\leq \tilde{\mu }{\text{e}}^{\left(\frac{\text{ln}\tilde{\mu }}{\rho_{1}}-\omega \right)t}||\varepsilon \left(0\right)|{|}^{2}+\int_{0}^{t}\tilde{\mu }{\text{e}}^{\left(\frac{\text{ln}\tilde{\mu }}{\rho_{1}}-\omega \right)\left(t-s\right)}\\ \;\left[\beta_{1}\nu \left(s-\tau \left(s\right)\right)+\delta +\kappa \right]\text{d}s, \end{array}$$

that is,

(46) $$\begin{array}{l} \nu \left(t\right)\leq \zeta {\text{e}}^{\left(\frac{\text{ln}\tilde{\mu }}{\rho_{1}}-\omega \right)t}+\int_{0}^{t}{\text{e}}^{\left(\frac{\text{ln}\tilde{\mu }}{\rho_{1}}-\omega \right)\left(t-s\right)}\left[\tilde{\mu }\beta_{1}\nu \left(s-\tau \left(s\right)\right)+\tilde{\mu }\delta +\kappa \right]\text{d}s, \end{array}$$

where $\zeta =\tilde{\mu }\underset{\tau \leq t\leq 0}{\text{sup}}\left\{||\varepsilon \left(t\right)|{|}^{2}\right\}$.

Let $\ell \left(\rho \right)=2\rho +\frac{\text{ln}\tilde{\mu }}{\rho_{1}}-\omega +\tilde{\mu }\beta {\text{e}}^{2\rho \tau }$. For the continuous function ℓ(ρ), according to (21), ℓ(0) < 0, ℓ(+∞) > 0, and $\dot{\ell }\left(\rho \right)=2+2\tau \tilde{\mu }\beta {\text{e}}^{2\rho \tau }>0$. Besides, a unique solution ρ > 0 to $\dot{\ell }\left(\rho \right)=0$ exists. If −τ ≤ *t* ≤ 0, *r* > 0, $\tilde{\mu }\geq 1$, ρ > 0, and δ > 0 hold, we have

(47) $$\begin{array}{l} \zeta {\text{e}}^{\left(\frac{\text{ln}\tilde{\mu }}{\rho_{1}}-\omega \right)t}\leq \tilde{\mu }||\varepsilon \left(t\right)|{|}^{2}{\text{e}}^{\left(\frac{\text{ln}\tilde{\mu }}{\rho_{1}}+\varpi_{1}\right)t}\leq \tilde{\mu }||\epsilon \left(t\right)|{|}^{2}{\text{e}}^{-2\rho t}=\zeta {\text{e}}^{-2\rho t}. \end{array}$$

and

(48) $$\begin{array}{l} \begin{array}{l} \int_{0}^{t}{\text{e}}^{\left(\frac{\text{ln}\tilde{\mu }}{\rho_{1}}-\omega \right)\left(t-s\right)}\left[\tilde{\mu }\beta \nu \left(s-\tau \left(s\right)\right)+\tilde{\mu }\left(\delta +\kappa \right)\right]\text{d}s\\ \leq \int_{0}^{t}{\text{e}}^{\left(\frac{\text{ln}\tilde{\mu }}{\rho_{1}}-\omega \right)\left(t-s\right)}{\text{e}}^{\tilde{\mu }\beta \left(t-s\right)}\text{d}s+\int_{0}^{t}\tilde{\mu }\left(\delta +\kappa \right){\text{e}}^{\left(\frac{\text{ln}\tilde{\mu }}{\rho_{1}}-\omega \right)\left(t-s\right)}\text{d}s\\ =-\frac{1}{\frac{\text{ln}\tilde{\mu }}{\rho_{1}}-\omega +\tilde{\mu }\beta }+\frac{{\text{e}}^{\left(\frac{\text{ln}\tilde{\mu }}{\rho_{1}}-\omega +\tilde{\mu }\beta \right)}}{\frac{\text{ln}\tilde{\mu }}{\rho_{1}}-\omega +\tilde{\mu }\beta }-\frac{\tilde{\mu }\left(\delta +\kappa \right)}{\frac{\text{ln}\tilde{\mu }}{\rho_{1}}-\omega }\\ +\frac{\tilde{\mu }\left(\delta +\kappa \right)}{\frac{\text{ln}\tilde{\mu }}{\rho_{1}}-\omega }{\text{e}}^{\left(\frac{\text{ln}\tilde{\mu }}{\rho_{1}}-\omega \right)}\leq -\frac{1}{\frac{\text{ln}\tilde{\mu }}{\rho_{1}}-\omega +\tilde{\mu }\beta }-\frac{\tilde{\mu }\left(\delta +\kappa \right)}{\frac{\text{ln}\tilde{\mu }}{\rho_{1}}-\omega }\\ \leq \frac{\tilde{\mu }\left(\delta +\kappa \right)}{-\left(\frac{\text{ln}\tilde{\mu }}{\rho_{1}}-\omega \right)-\tilde{\mu }\beta }. \end{array} \end{array}$$

According to (22), taking (47) and (48) into account, for *t* > 0, the following inequality holds

(49) $$\begin{array}{l} \upsilon \left(t\right)<\zeta {\text{e}}^{-2\rho t}+\frac{\tilde{\mu }\left(\delta +\kappa \right)}{-\left(\frac{\text{ln}\tilde{\mu }}{\rho_{1}}-\omega \right)-\tilde{\mu }\beta } \end{array}$$

For *t* > 0, Equation (49) will be testified. Thus, if Equation (49) does not hold, there is t^*^ > 0 such that

(50) $$\begin{array}{l} \nu \left(t^{*}\right)\geq \zeta {\text{e}}^{-2\rho t^{*}}+\frac{\tilde{\mu }\left(\delta +\kappa \right)}{-\left(\frac{\text{ln}\tilde{\mu }}{\rho_{1}}-\omega \right)-\tilde{\mu }\beta }, \end{array}$$

and

(51) $$\begin{array}{l} \nu \left(t\right)<\zeta {\text{e}}^{-2\rho t}+\frac{\tilde{\mu }\left(\delta +\kappa \right)}{-\left(\frac{\text{ln}\tilde{\mu }}{\rho_{1}}-\omega \right)-\tilde{\mu }\beta },t<t^{*}. \end{array}$$

Combining Equations (50) and (51), there is

(52) $$\begin{array}{l} \begin{array}{l} \;\nu \left(t^{*}\right)\leq \zeta {\text{e}}^{\left(\frac{\text{ln}\tilde{\mu }}{\rho_{1}}-\omega \right)t^{*}}+\int_{0}^{t^{*}}{\text{e}}^{\left(\frac{\text{ln}\tilde{\mu }}{\rho_{1}}-\omega \right)\left(t^{*}-s\right)}\\ \;\left[\tilde{\mu }\beta \upsilon \left(s-\tau \left(s\right)\right)+\tilde{\mu }\left(\delta +\kappa \right)\right]\text{d}s,\\ \;<{\text{e}}^{\left(\frac{\text{ln}\tilde{\mu }}{\rho_{1}}-\omega \right)t^{*}}\{\zeta +\frac{\tilde{\mu }\left(\delta +\kappa \right)}{-\left(\frac{\text{ln}\tilde{\mu }}{\rho_{1}}-\omega \right)-\tilde{\mu }\beta }\\ \;+\int_{0}^{t^{*}}{\text{e}}^{-\left(\frac{\text{ln}\tilde{\mu }}{\rho_{1}}-\omega \right)s}\left[\tilde{\mu }\beta \upsilon \left(s-\tau \left(s\right)\right)+\tilde{\mu }\left(\delta +\kappa \right)\right]\text{d}s\}. \end{array} \end{array}$$

When *t* < t^*^, there is

(53) $$\begin{array}{l} \nu \left(t\right)<\zeta {\text{e}}^{-2\rho t}+\frac{\tilde{\mu }\left(\delta +\kappa \right)}{-\left(\frac{\text{ln}\tilde{\mu }}{\rho_{1}}-\omega \right)-\tilde{\mu }\beta }. \end{array}$$

Because *s* ∈ (0, t^*^), therefore we obtain

(54) $$\begin{array}{l} \nu \left(s-\tau \left(s\right)\right)<\zeta {\text{e}}^{-2\rho \left(s-\tau \left(s\right)\right)}+\frac{\tilde{\mu }\left(\delta +\kappa \right)}{-\left(\frac{\text{ln}\tilde{\mu }}{\rho_{1}}-\omega \right)-\tilde{\mu }\beta }. \end{array}$$

Then, we have the following conclusion

(55) $$\begin{array}{l} \begin{array}{l} \nu \left(t^{*}\right)\leq \zeta {\text{e}}^{\left(\frac{\text{ln}\tilde{\mu }}{\rho_{1}}-\omega \right)t^{*}}\{\zeta +\frac{\tilde{\mu }\left(\delta +\kappa \right)}{-\left(\frac{\text{ln}\tilde{\mu }}{\rho_{1}}-\omega \right)-\tilde{\mu }\beta }\\ +\int_{0}^{t^{*}}{\text{e}}^{-\left(\frac{\text{ln}\tilde{\mu }}{\rho_{1}}-\omega \right)s}\left[\tilde{\mu }\beta \left(\zeta {\text{e}}^{-2\zeta \left(s-\tau \left(s\right)\right)}+\frac{\tilde{\mu }\left(\delta +\kappa \right)}{-\left(\frac{\text{ln}\tilde{\mu }}{\rho_{1}}-\omega \right)-\tilde{\mu }\beta }\right)+\tilde{\mu }\left(\delta +\kappa \right)\right]\text{d}s\}\\ \leq \nu \left(t^{*}\right)\zeta {\text{e}}^{\left(\frac{\text{ln}\tilde{\mu }}{\rho_{1}}-\omega \right)t^{*}}\{\zeta +\frac{\tilde{\mu }\left(\delta +\kappa \right)}{-\left(\frac{\text{ln}\tilde{\mu }}{\rho_{1}}-\omega \right)-\tilde{\mu }\beta }\\ +\frac{\tilde{\mu }\left(\delta +\kappa \right)\beta }{-\left(\frac{\text{ln}\tilde{\mu }}{\rho_{1}}-\omega +2\rho \right)}{\text{e}}^{2\rho \tau }\left[{\text{e}}^{-\left(\frac{\text{ln}\tilde{\mu }}{\rho_{1}}-\omega +2\rho \right)t^{*}}-1\right]\\ +\frac{\tilde{\mu }\left(\delta +\kappa \right)}{-\left(\frac{\text{ln}\tilde{\mu }}{\rho_{1}}-\omega \right)-\tilde{\mu }\beta }\left[{\text{e}}^{-\left(\frac{\text{ln}\tilde{\mu }}{\rho_{1}}-\omega \right)t^{*}}-1\right]\}\\ =\zeta {\text{e}}^{-2\rho t^{*}}+\frac{\tilde{\mu }\left(\delta +\kappa \right)}{-\left(\frac{\text{ln}\tilde{\mu }}{\rho_{1}}-\omega \right)-\tilde{\mu }\beta }. \end{array} \end{array}$$

It can be seen that Equation (55) contradicts Equation (50). Then, Equation (49) holds for *t* > 0. When δ > 0, based on Equation (41), there is

(56) $$\begin{array}{l} V\left(t\right)\leq \nu \left(t\right)\leq \zeta {\text{e}}^{-2\rho t^{*}}+\frac{\tilde{\mu }\kappa }{-\left(\frac{\text{ln}\tilde{\mu }}{\rho_{1}}-\omega \right)-\tilde{\mu }\beta },t>0. \end{array}$$

Setting δ → 0, according to Equation (55), there is

(57) $$\begin{array}{l} V\left(t\right)\leq \nu \left(t\right)\leq \zeta {\text{e}}^{-2\rho t}=\tilde{\mu }\underset{\tau \leq s\leq 0}{\text{max}}\left\{||\psi_{1}\left(t\right)|{|}^{2}{\text{e}}^{-2\rho t}\right\}. \end{array}$$

Combining Equation (27) and Equation (55), we have

(58) $$\begin{array}{l} \begin{array}{l} ||\varepsilon \left(t\right)||\leq \sqrt{\zeta {\text{e}}^{-2\rho t}+\frac{\tilde{\mu }\kappa }{-\left(\frac{\text{ln}\tilde{\mu }}{\rho_{1}}-\omega \right)-\tilde{\mu }\beta }}\\ \;\leq \sqrt{\frac{\tilde{\mu }\kappa }{-\left(\frac{\text{ln}\tilde{\mu }}{\rho_{1}}-\omega \right)-\tilde{\mu }\beta }}+\sqrt{\zeta }{\text{e}}^{-\rho t},t\geq 0. \end{array} \end{array}$$

Consider Definition 1, the projective quasi-synchronization of systems (8) and (9) is realized by the event-triggered control method. For the error system (12), its trajectory exponentially converges into the compact set 𝔻 with a convergence rate ρ of *t* → +∞. 𝔻 can be expressed as

(59) $$\begin{array}{l} 𝔻=\left\{\varepsilon \left(t\right)\in R^{N}|||\varepsilon \left(t\right)||\leq \sqrt{\frac{\tilde{\mu }\kappa }{-\left(\frac{\text{ln}\tilde{\mu }}{\rho_{1}}-\omega \right)-\tilde{\mu }\beta }}\right\} \end{array}$$

The proof is complete.

**Remark 3**. It should be noticed that continuous communication between the drive-response systems is always required to monitor the triggered condition (25). Therefore, the self-triggered method is designed to solve this problem.

Then, this article investigates the lower bound of inter-execution time to eliminate the Zeno behavior for the error system (12). The following theorem indicates that the system avoids the Zeno behavior under the bound of *t*_*k*_ − *t*_*k*−1_ > 0.

**Theorem 2**. *The triggered instants t*_*k*_(*k* ∈ *N*_+_) *can be calculated under the error system (12) and control method (11). Meanwhile, if there is a positive constant ρ*_1_ *that satisfies* $\underset{k\in N_{+}}{\text{inf}}${*t*_*k*_ − *t*_*k*−1_} ≥ *ρ*_1_ > 0, *then the error system (12) can avoid Zeno behaviors*.

*Proof:* For [*t*_*k*_, *t*_*k*−1_), according to Assumptions 1–2, there is

(60) $$\begin{array}{l} \begin{array}{l} D^{+}||e\left(t\right)||\leq ||ė\left(t\right)||=||\dot{\varepsilon }\left(t\right)||\\ \leq ||-\tilde{D}\left(y\left(t\right)\right)\varepsilon \left(t\right)+\left(\tilde{A}\left(y\left(t\right)\right)+\Delta A^{*}\left(t\right)\right)f\left(\varepsilon \left(t\right)\right)\\ +(\tilde{B}\left(y\left(t\right)\right)+\Delta B^{*}\left(\left(t-\tau \left(t\right)\right)\right)f\left(\varepsilon \left(t-\tau \left(t\right)\right)\right)+\alpha \sigma \Gamma W\varepsilon \left(t\right)\\ +\text{Ψ}\left(t\right)+K\varepsilon \left(t_{k-1}\right)||\\ \leq \left[\lambda_{\text{max}}\left(\bar{D}\right)+\lambda_{\text{max}}\left(F\right)\lambda_{\text{max}}\left(Ā+A^{*}\right)+\sigma \lambda_{\text{max}}\left(\Gamma \right)\lambda_{\text{max}}\left(W\right)\right]\\ \times ||e(t||+[\lambda_{\text{max}}\left(\bar{D}\right)+\lambda_{\text{max}}\left(F\right)\lambda_{\text{max}}\left(Ā+A^{*}\right)\\ +\sigma \lambda_{\text{max}}\left(\Gamma \right)\lambda_{\text{max}}\left(W\right)+\lambda_{\text{max}}\left(K\right)]||\varepsilon \left(t_{k}-1\right)||+||\Gamma_{4}||, \end{array} \end{array}$$

where $K=\text{diag}\left\{\left|K_{1}|,|K_{2}|,\ldots ,|K_{N}\right|\right\}$, and $\Gamma_{4}=\left[\left(2\alpha +1\right)\lambda_{\text{max}}\left(\bar{B}\right)+\lambda_{\text{max}}\left(A\right)+\left(\alpha +1\right)\lambda_{\text{max}}\left(Ā+B^{*}\right)+\alpha \lambda_{\text{max}}\left(B+A^{*}\right)\right]\lambda_{\text{max}}\left(M\right)$.

Then, according to *e*(*t*_*k*−1_) = 0, there is

(61) $$\begin{array}{l} ||e\left(t\right)||\leq \frac{\Xi_{1}}{\Xi_{2}}\times \left[\text{exp}\left(\Xi_{2}\left(t-t_{k-1}\right)\right)-1\right], \end{array}$$

and $\Xi_{1}=\left[\lambda_{\text{max}}\left(\bar{D}\right)+\lambda_{\text{max}}\left(F\right)\lambda_{\text{max}}\left(Ā+A^{*}\right)+\sigma \lambda_{\text{max}}\left(\Gamma \right)\lambda_{\text{max}}\left(W\right)+\lambda_{\text{max}}\left(K\right)\right]||\varepsilon \left(t_{k}-1\right)||+||\Gamma_{4}||$, $\Xi_{2}=\lambda_{\text{max}}\left(\bar{D}\right)+\lambda_{\text{max}}\left(F\right)\lambda_{\text{max}}\left(Ā+A^{*}\right)+\sigma \lambda_{\text{max}}\left(\Gamma \right)\lambda_{\text{max}}\left(W\right)$.

According to the event-triggered condition (25), we have

(62) $$\begin{array}{l} \sqrt{\ell \left(\varepsilon \left(t\right)\right)}\leq \frac{\Xi_{1}}{\Xi_{2}}\times \left[\text{exp}\left(\Xi_{2}\left(t-t_{k-1}\right)\right)-1\right], \end{array}$$

and $\ell \left(\varepsilon \left(t\right)\right)=-2\alpha_{2}|\varepsilon \left(t\right)|\left(\tilde{\alpha }-\frac{1}{2}\alpha^{2}\alpha_{1}-\frac{1}{2}\alpha_{2}\Gamma_{3}^{\text{T}}\Gamma_{3}-\frac{1}{2}\alpha K^{2}\right)|\varepsilon \left(t\right)|$.

For *T*_*k*−1_ = *t*_*k*_ − *t*_*k*−1_, *t* ∈ [*t*_*k*−1_, *t*_*k*_), *k* ∈ *N*_+_, there is

(63) $$\begin{array}{l} T_{k-1}\geq \frac{1}{\Xi_{2}}\text{ln}\left(1+\frac{\Xi_{2}}{\Xi_{1}}\sqrt{\ell \left(\varepsilon \left(t\right)\right)}\right). \end{array}$$

Thus, it can be derived that there is a lower bound of the inter-execution time, and *T*_*k*−1_ = *t*_*k*_ − *t*_*k*−1_ > 0. The error system (12) can avoid Zeno behaviors.

The completion of the proof is shown above.

Although the systems between (8) and (9) can achieve projective quasi-synchronization, the triggered condition (25) is always monitored during the continuous communication process. To address this issue, this article develops a self-triggered mechanism to update the trigger sequence without monitoring the triggered condition. In this way, there is no need to use the self-triggered mechanism to obtain the state information continuously from Theorem 2 if *T*_*k*−1_ satisfies Equation (63). Thus, the triggered instant should satisfy the following condition:

(64) $$\begin{array}{l} {\tilde{t}}_{k}={\tilde{t}}_{k-1}+\frac{1}{\Xi_{2}}\text{ln}\left(1+\frac{\Xi_{2}}{\Xi_{1}}\sqrt{\ell \left(\varepsilon \left(t\right)\right)}\right). \end{array}$$

The sampling and control instants are calculated by Equation (64) during the self-triggered process as shown in self-triggering Algorithm 1. It is demonstrated that the instants of the controller will not update if the triggered condition is more comprehensive than the second term of Equation (64).

**Algorithm 1:** Self-triggered algorithm.

**Require:***t* = *t*_0_, *k* ∈ *N*_+_, *t*_*k*−1_ = *t*_0_
**Ensure:**$\tilde{D}$,Ã,$\tilde{B}$,Ξ_1_,Ξ_2_
1: **for** *l, m* to *n* **do**
2: // Determine $\tilde{D}$, Ã, and $\tilde{B}$ by (4) using initial values *x*(*t*_0_) and *y*(*t*_0_);
3: **end for**
4: // Denote ε(*t*) = ε(*t*_*k*−1_)
5: **while** *t* < *T*, // *T* is the complete time of the entire system. **do**
6: **for** *l, m* to *n* **do**
7: // Determine $\tilde{D}$, Ã, and $\tilde{B}$ by (4) using initial values *x*(*t*_*k*−1_) and *y*(*t*_*k*−1_);
8: **end for**
9: // Compute Ξ_1_ and Ξ_2_,
10: // ${\tilde{t}}_{k}={\tilde{t}}_{k-1}+\frac{1}{\Xi_{2}}\text{ln}\left(1+\frac{\Xi_{2}}{\Xi_{1}}\sqrt{\ell \left(\varepsilon \left(t\right)\right)}\right)$
11: **if** $t={\tilde{t}}_{k}$ // which means the system is triggered. **then**
12: // Update *k* = *k* + 1; *t*_*k*−1_ = *t*; ε(*t*) = ε(*t*_*k*−1_)
13: **end if**
14: **end while**

**Theorem 3**. *Combining the control method (11) and error system (12) and using the self-triggered mechanism, the drive and response systems (8) and (9) can achieve projective quasi-synchronization according to the triggered sequence* $\left\{{\tilde{t}}_{k}^{\infty }\right\}$ *produced by Equation (64). Meanwhile, the error system (12) can avoid the Zeno behavior*.

*Proof:* The self-triggered instants meet Equation (64). According to Equation (63), we have $t_{k}\geq {\tilde{t}}_{k}$. Then, the event-triggered condition is illustrated in Equation (64). Meanwhile, we have ${\tilde{t}}_{k-1}<{\tilde{t}}_{k}$ because $\frac{1}{\Xi_{2}}\text{ln}\left(1+\frac{\Xi_{2}}{\Xi_{1}}\sqrt{\ell \left(\varepsilon \left(t\right)\right)}\right)>0$ always holds. Therefore, the self-triggered mechanism Equation (64) guarantees the projective quai-synchronization between (8) and (9) through (11). Also, the error system (12) avoids the Zeno behavior. The proof is completed.

## 4. Numerical simulation

### 4.1. Conclusions proof

Numerical simulations are conducted to verify the correctness of the theoretical analysis results. Systems (8) and (9) including three (*N* = 3) nodes are considered. Each node has three (*n* = 3) dimensions of information. Additionally, *x*_11_ means the 1*st* dimension of Node 1. For the coupling framework, the coupling strength σ = 1.5; the inner connecting matrix Γ and coupled matrix *W* are set as

$$\Gamma =\left[\begin{array}{ccc} 1 & 0 & 0\\ 0 & 1 & 0\\ 0 & 0 & 1 \end{array}\right],\text{W}={\left(w_{ij}\right)}_{3\times 3}=\left[\begin{array}{ccc} -2 & 1 & 1\\ 1 & -1 & 0\\ 1 & 0 & -1 \end{array}\right].$$

For systems (8) and (9), the time-varying delay can be represented as τ(*t*) = e^*t*^/(e^*t*^ + 1). Considering Assumptions 1 and 2, this article sets *L*_*f*_ = 1 and *M*_*i*_ = 1(*i* = 1, 2, 3). In this example, the following initial values are taken: $x_{1}\left(0\right)={\left[-1.55,1.05,5.12\right]}^{\text{T}}$, $x_{2}\left(0\right)={\left[0.35,-6.98,0.34\right]}^{\text{T}}$, $x_{3}\left(0\right)={\left[3.25,-1.15,7.1\right]}^{\text{T}}$, $y_{1}\left(0\right)={\left[0.82,-0.43,-0.53\right]}^{\text{T}}$, $y_{2}\left(0\right)={\left[0.09,-0.58,-0.42\right]}^{\text{T}}$, $y_{3}\left(0\right)={\left[-1.67,-0.88,0.15\right]}^{\text{T}}$.

The parameters of the systems are as follows:

$$\text{D}=\left[\begin{array}{ccc} 1.5 & 1 & 1\\ 1.3 & 1.2 & 1\\ 1.2 & 1 & 1 \end{array}\right],{\bar{\text{A}}}_{1}=\left[\begin{array}{ccc} 1.8 & 2.8 & 2.9\\ 1.3 & 1.7 & 1.6\\ 1.2 & 2.0 & 1.4 \end{array}\right],{\bar{\text{A}}}_{2}=\left[\begin{array}{ccc} 1.8 & 2.7 & 2.0\\ 1.4 & 1.5 & 1.4\\ 1.2 & 2.0 & 1.4 \end{array}\right],$$

$${\bar{\text{A}}}_{3}=\left[\begin{array}{ccc} 2.0 & 3.0 & 0.9\\ 1.5 & 1.9 & 1.5\\ 1.3 & 2.1 & 1.1 \end{array}\right],{\bar{\text{B}}}_{1}=\left[\begin{array}{ccc} 3.8 & 3.0 & 0.9\\ 0.4 & 0.3 & 0.6\\ 3.4 & 3.8 & 1.9 \end{array}\right],$$

$${\bar{\text{B}}}_{2}=\left[\begin{array}{ccc} 3.7 & 2.8 & 2.9\\ 0.5 & 0.4 & 0.9\\ 3.2 & 3.1 & 2.0 \end{array}\right],{\bar{\text{B}}}_{3}=\left[\begin{array}{ccc} 3.5 & 2.8 & 1.2\\ 0.6 & 0.4 & 0.9\\ 3.4 & 3.8 & 1.9 \end{array}\right],$$

The uncertainties of the proposed systems are

$$\Delta \text{A}=0.1\text{sin}\left(t\right)\left[\begin{array}{ccc} 1 & 1 & 1\\ 1 & 1 & 1\\ 1 & 1 & 1 \end{array}\right],\Delta \text{B}=-0.2\text{sin}\left(t-\tau \left(t\right)\right)\left[\begin{array}{ccc} 1 & 1 & 1\\ 1 & 1 & 1\\ 1 & 1 & 1 \end{array}\right].$$

$$\Delta {\text{A}}^{*}=0.2\text{tanh}\left(t\right)\left[\begin{array}{ccc} 1 & 1 & 1\\ 1 & 1 & 1\\ 1 & 1 & 1 \end{array}\right],\Delta {\text{B}}^{*}=-0.2\text{tanh}\left(t-\tau \left(t\right)\right)\left[\begin{array}{ccc} 1 & 1 & 1\\ 1 & 1 & 1\\ 1 & 1 & 1 \end{array}\right].$$

The nonlinear activation function *F*(*x*_*i*_(*t*))(*i* = 1, 2, 3) is given as:

$$\text{F}{(\text{x}}_{\text{i}}\left(\text{t})\right)=\left[\begin{array}{c} f_{1}\left(x_{i_{1}}\left(t\right)\right)\\ f_{2}\left(x_{i_{2}}\left(t\right)\right)\\ f_{3}\left(x_{i_{3}}\left(t\right)\right) \end{array}\right]=\left[\begin{array}{c} \frac{\left|x_{i_{1}}+1|-|x_{i_{1}}-1\right|}{2}-1\\ \frac{\left|x_{i_{2}}+1|-|x_{i_{2}}-1\right|}{2}-1\\ \frac{\left|x_{i_{3}}+1|-|x_{i_{3}}-1\right|}{2}-1 \end{array}\right],$$

**Figure 3:**
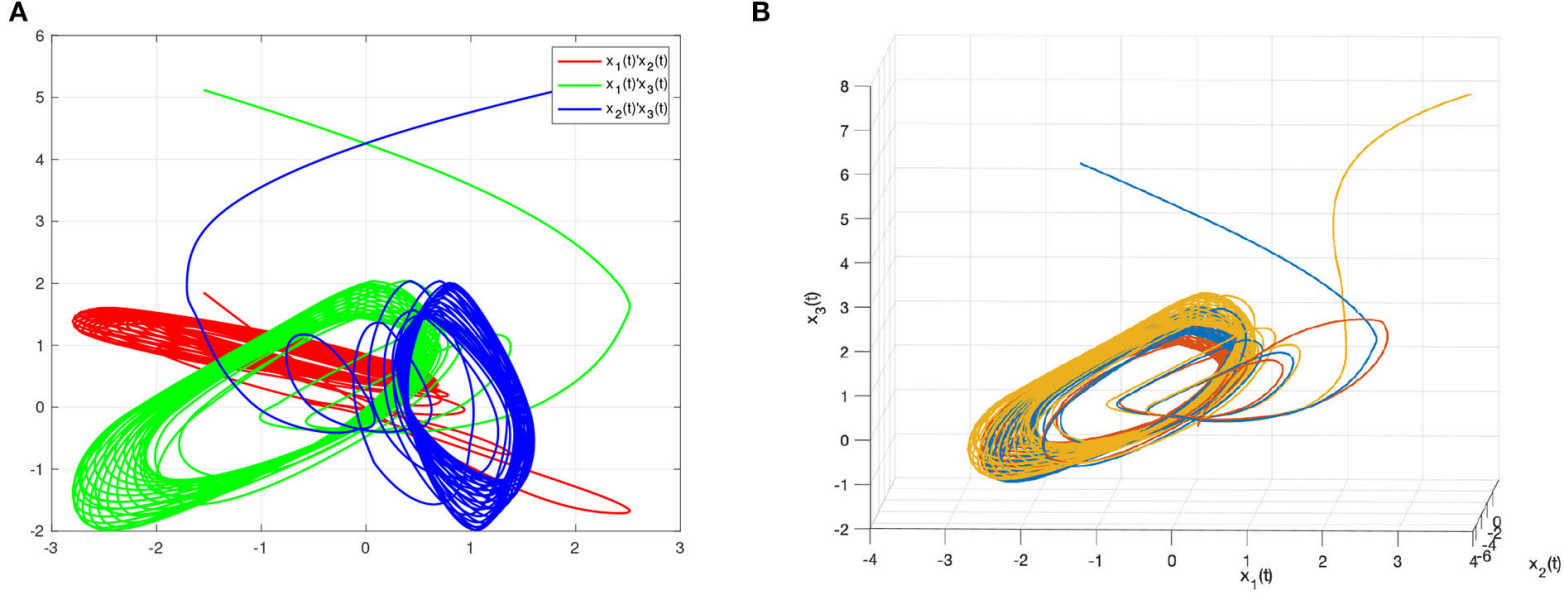
The chaotic dynamic behavior of systems (8) and (9). **(A)** Time evolutions of systems in a complex space. **(B)** State curves of systems in a complex plane.

**Figure 4:**
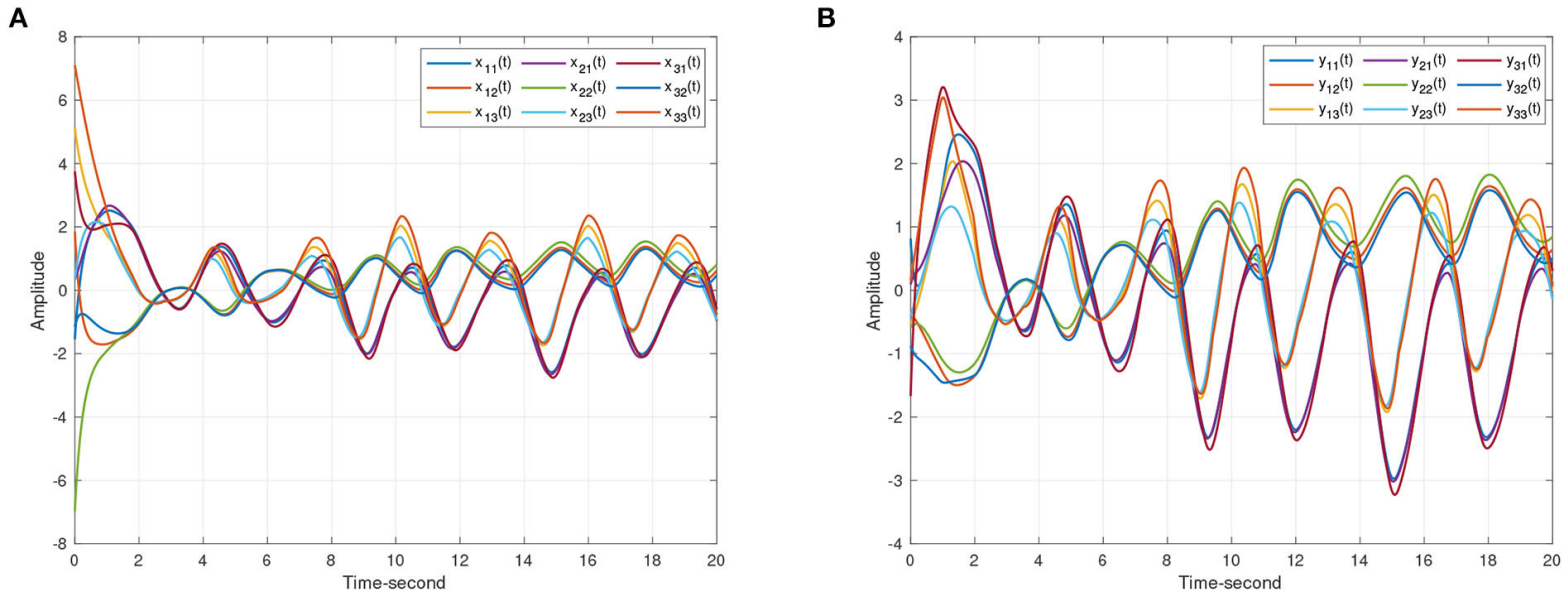
The state trajectories produced by systems (8) and (9) without the controller. **(A)** The state curves of drive system (8). **(B)** The state curves of response system (9).

**Figure 5:**
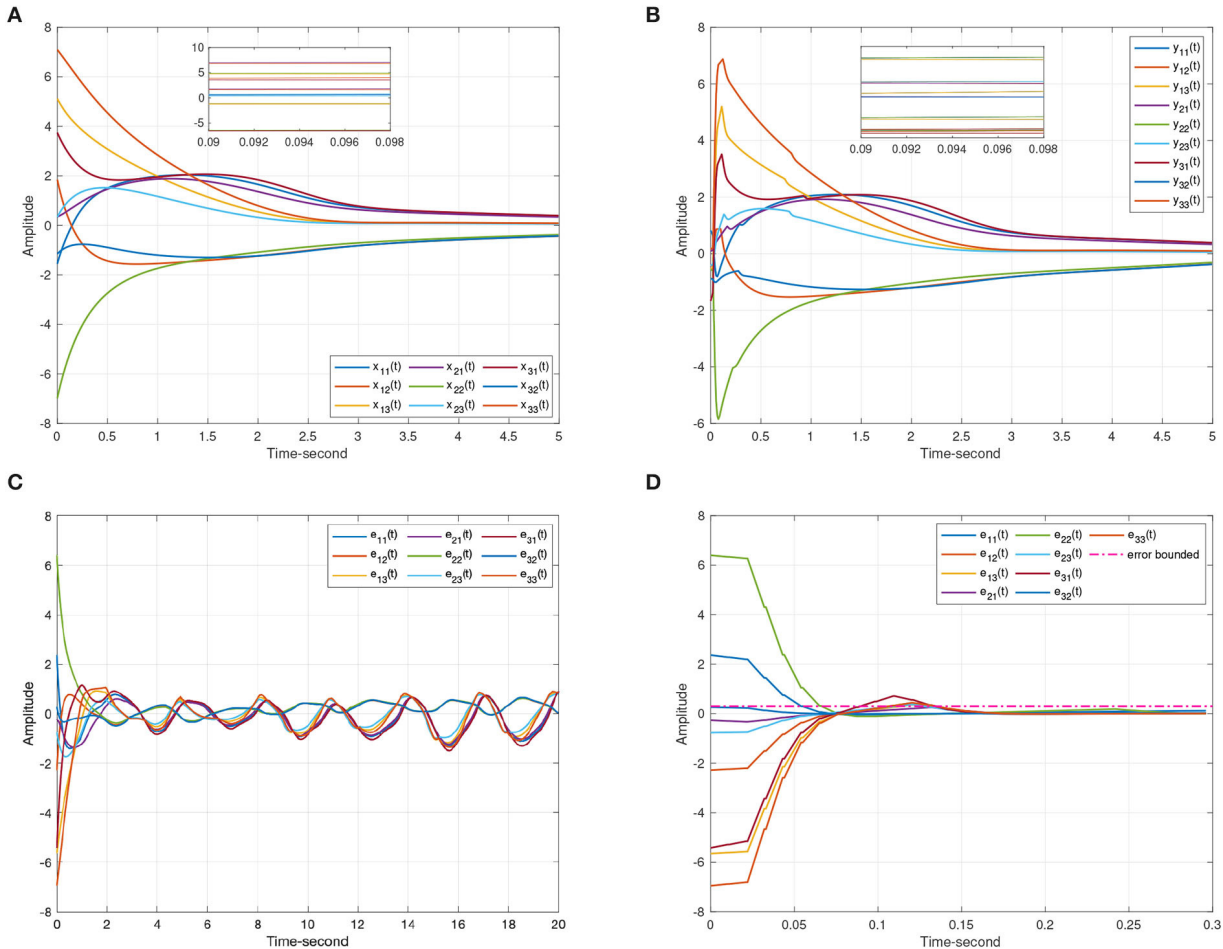
The synchronization state trajectories produced by systems (8) and (9). **(A)** The state curves of the drive system (8). **(B)** The state curves of the response system (9). **(C)** The synchronization errors ε(*t*) = *y*(*t*) − *x*(*t*) without control. **(D)** The synchronization errors ε(*t*) = *y*(*t*) − *x*(*t*) under the event-triggered scheme (11).

As discussed above, Figure 3 represents the chaotic behavior of (8) and (9) under different initial conditions. And Figure 4 shows the states of (8) and (9) without controller. Then, this article defines the projector factor α = 1, i.e., the synchronization error ε(*t*) = *y*(*t*) − *x*(*t*). For Lemma 4, this article sets α_1_ = α_2_ = α_3_ = 0.01. Figure 5 shows the trajectory of (8) and (9) without control.

The gain matrix of the controller is

$$K=30\left[\begin{array}{ccc} 1 & 1 & 1\\ 1 & 1 & 1\\ 1 & 1 & 1 \end{array}\right].$$

This article chooses μ_*k*_ = 0.9, and it can easily testify that all the rules in Theorem 1 hold, which indicates that systems (8) and (9) can achieve quasi-synchronization with the projective factor α = 1.

**Figure 6:**
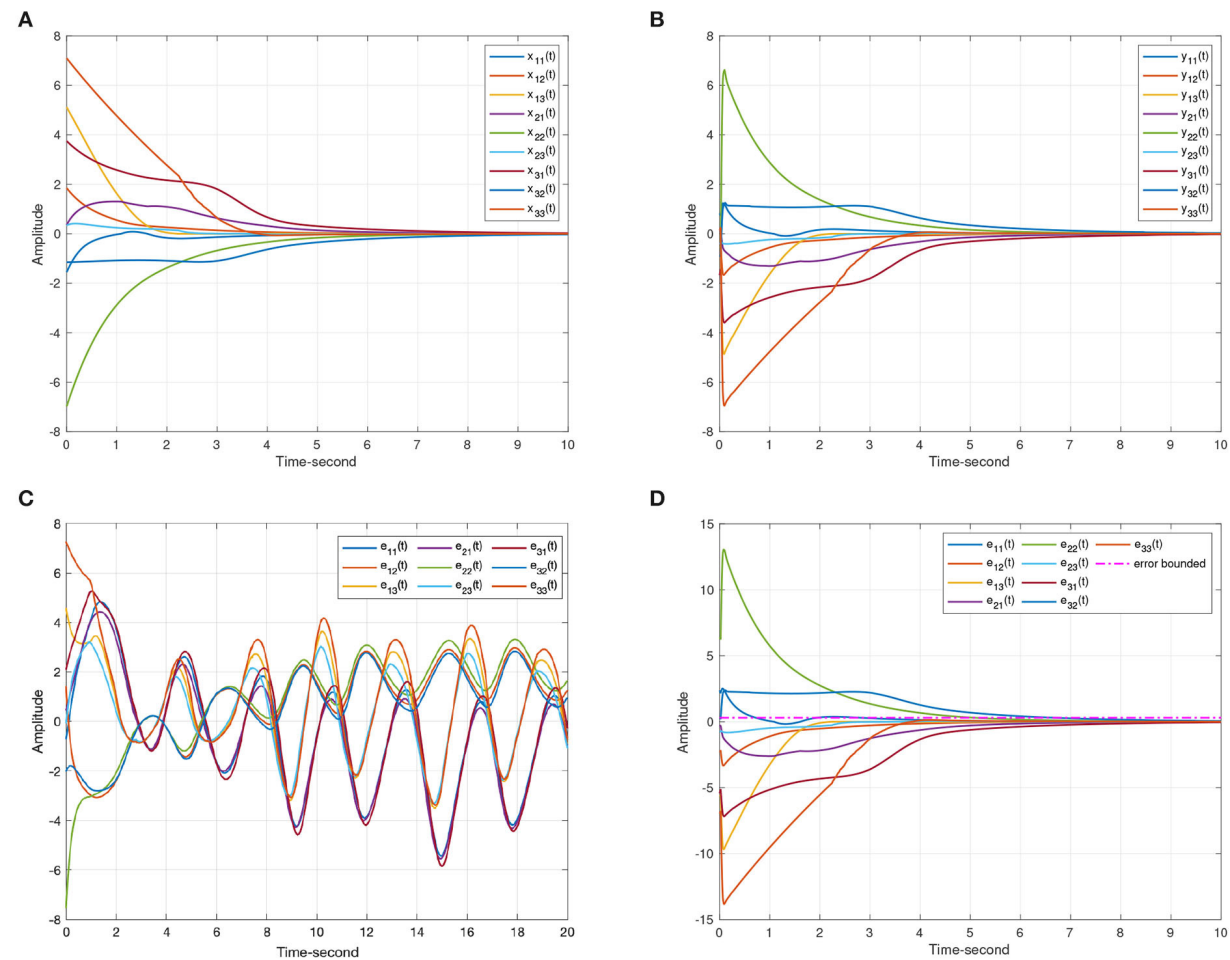
The anti-synchronization state trajectories produced by systems (8) and (9). **(A)** The state curves of the drive system (8). **(B)** The state curves of the response system (9). **(C)** The anti-synchronization errors ε(*t*) = *y*(*t*) + *x*(*t*). **(D)** The anti-synchronization errors ε(*t*) = *y*(*t*) + *x*(*t*) under the event-triggered scheme (11).

When the projective factor α = −1, anti quai-synchronization can be also achieved between systems (8) and (9). Considering Lemma 4, this article sets α_1_ = 1000, α_2_ = 1000, α_3_ = 0.01. Figure 6 shows the effectiveness of Theorem 1.

**Figure 7:**
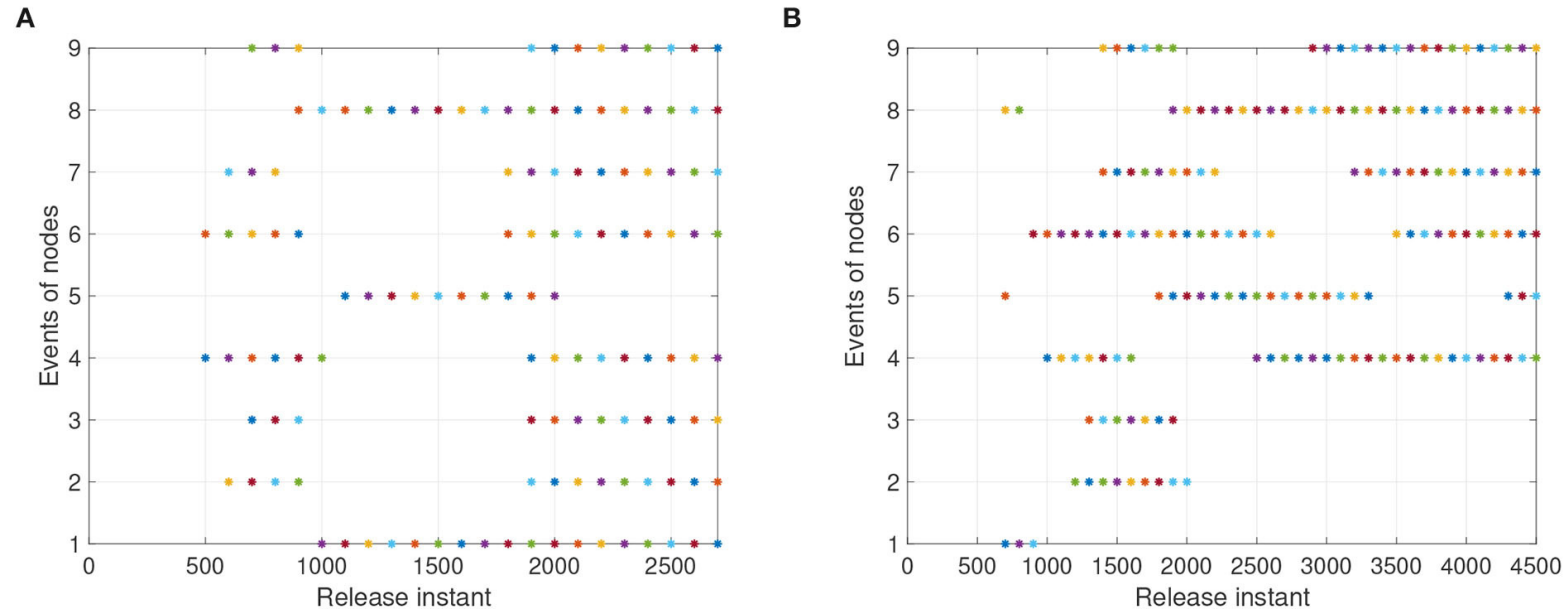
The events with event-triggered control. **(A)** Instants for synchronization ε(*t*) = *y*(*t*) − *x*(*t*). **(B)** Instants for anti-synchronization ε(*t*) = *y*(*t*) + *x*(*t*).

**Figure 8:**
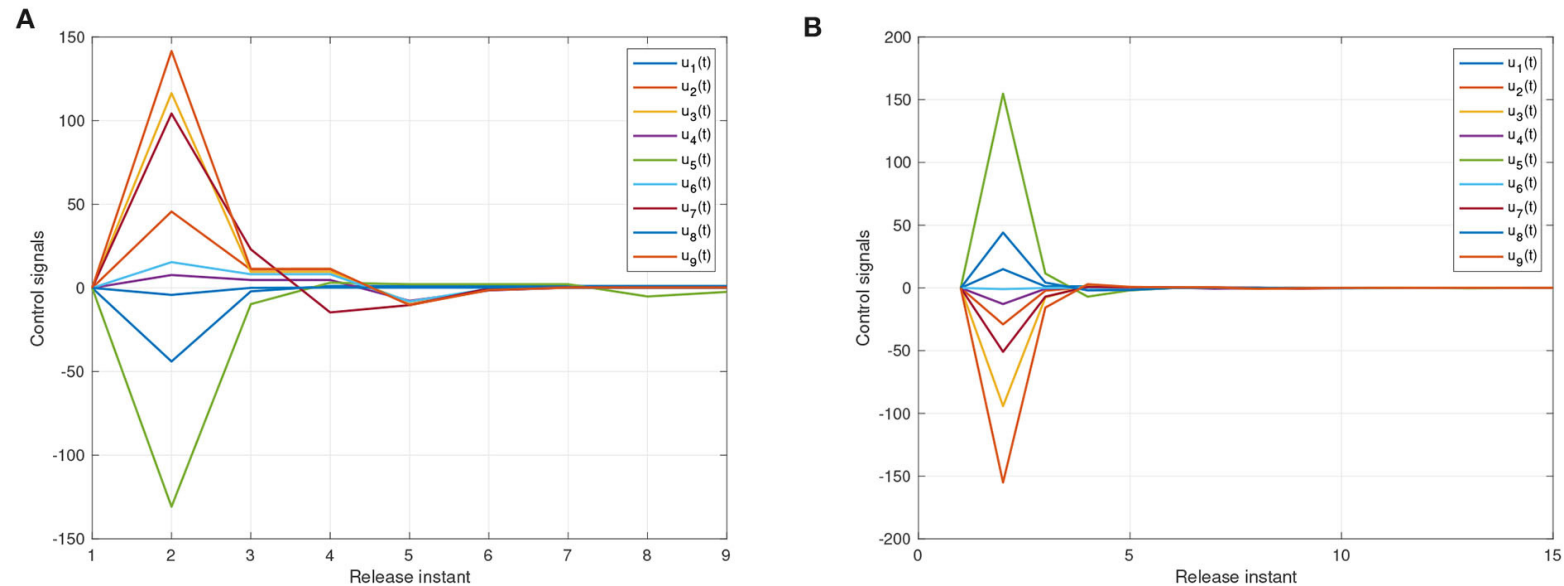
The states of event-triggered controller (11). **(A)** Instants for synchronization ε(*t*) = *y*(*t*) − *x*(*t*). **(B)** Instants for anti-synchronization ε(*t*) = *y*(*t*) + *x*(*t*).

**Figure 9:**
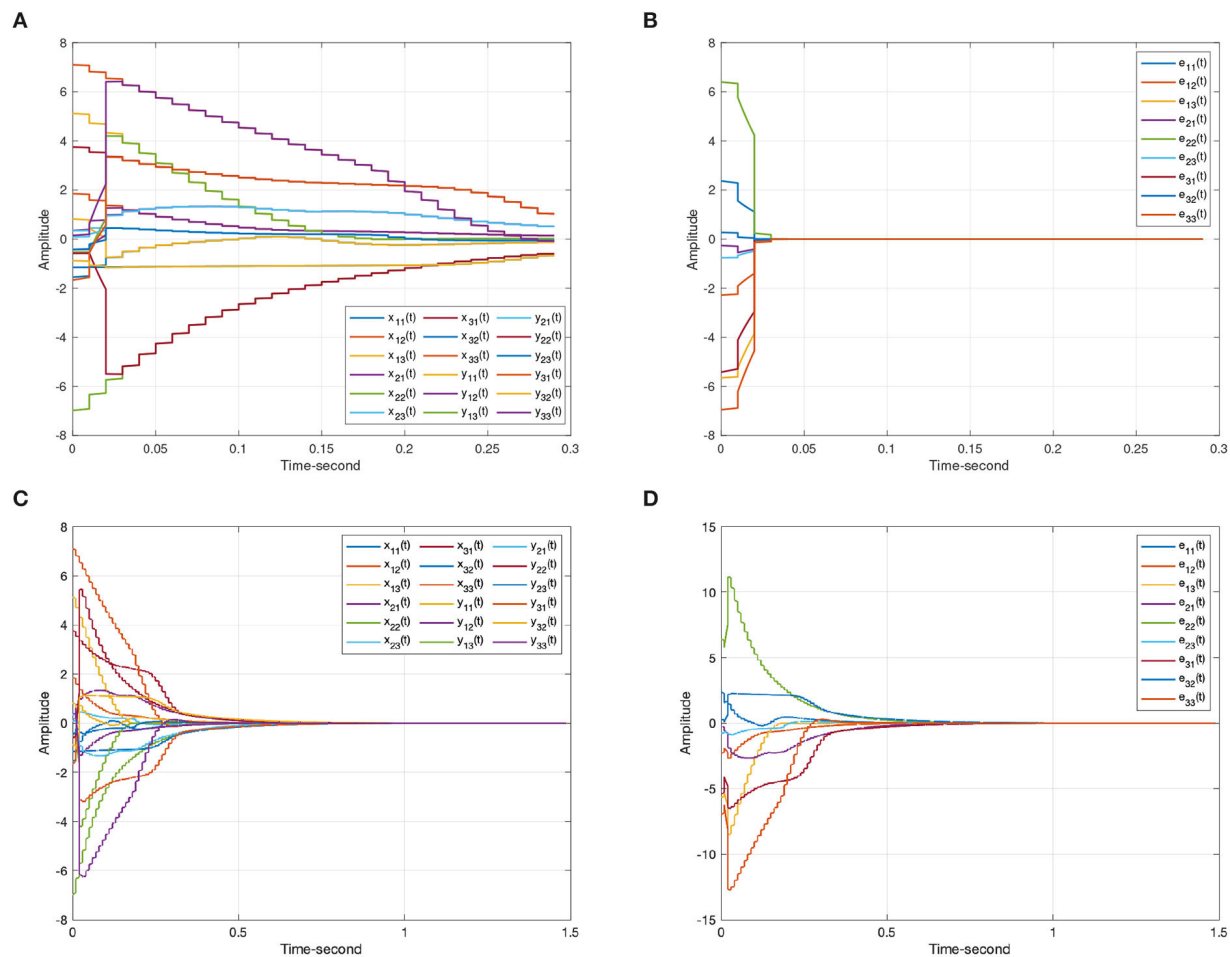
The state trajectories produced by systems (8) and (9) under a self-triggered scheme (11). **(A)** The state curves of systems (8) and (9) with α = 1. **(B)** The synchronization errors ε(*t*) = *y*(*t*) − *x*(*t*). **(C)** The state curves of systems (8) and (9) with α = −1. **(D)** The anti-synchronization errors ε(*t*) = *y*(*t*) + *x*(*t*).

**Figure 10:**
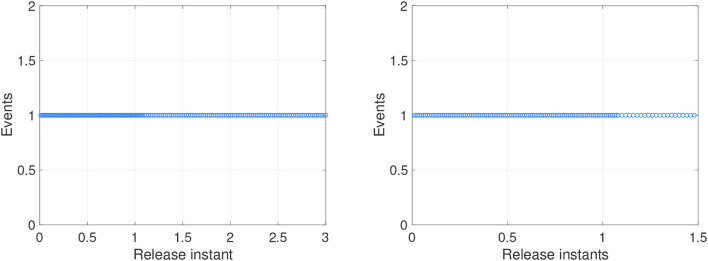
The events with self-triggered control. **(A)** Instants for synchronization ε(*t*) = *y*(*t*) − *x*(*t*). **(B)** Instants for anti-synchronization ε(*t*) = *y*(*t*) + *x*(*t*).

For systems (8) and (9), Figures 5, 6 represent the state trajectory and the synchronization error under event-triggered control for different projective factors. Also, the synchronization error norm is illustrated. Figures 7, 8 present the state-feedback controller and events under event-triggered control. Figures 9, 10 demonstrate the performance of the self-triggered scheme. The comparison between Figures 7, 10 indicates that the lower bound of the event-triggered time interval is set to the update time for the self-triggered scheme. Therefore, the self-triggered scheme has a higher update frequency than the event-triggered one. Meanwhile, the information exchange and deterioration are increased within limited network resources.

### 4.2. Existing achievements comparison

Now, we make our proposed event-triggered mechanism of coupled MNNs compared with some exciting methods to illustrate our designed method's superiority in synchronization, especially in the situation of limited bandwidth. Due to the different types of neural networks, we set the interval as [0.0, 1.0] to conduct the contrast simulations as shown in Table 1, in which the mean time interval means the average update frequency between each trigger. It is noticed that the lowest value of our method is 0.013 s, which indicates the control input can complete more than 76.92 updates/s in general.

**Table 1:** Performance comparison of event-triggered examples.

**Control method**	**Mean time interval**			
	**1**	**2**	**3**	**4**
Wang W. et al. (2021)	0.0198	0.0198	0.0198	–
Yang et al. (2018)	0.0127	0.0119	0.0151	–
Liu J. et al. (2020)	0.0114	0.0116	0.0117	0.0046
Liu et al. (2019)	0.0153	0.0114	0.0160	0.0214
Theorem 1 (Synchronization)	0.0140	0.0131	0.0215	–
Theorem 1(Anti-synchronization)	0.1008	0.2481	0.1645	–

**Remark 4**. Compared with some existing results (Yang et al., 2018; Liu et al., 2019; Liu J. et al., 2020; Wang W. et al., 2021), it can be seen that our event-triggered method relies on fewer triggering events from Table 1. Meanwhile, it is less likely to trigger than other methods, indicating that, in the control process, our method consumes remarkably less energy for data calculation and detection.

The proposed method in this article is the event-triggered scheme, which means the control input information exchange and update which depends on the designed triggered function. When the proposed method did not work, the proposed scheme will become the traditional time-sampled scheme, and the control updating instants just rely on the fixed interval. Therefore, the proposed method is more general and flexible than other methods, especially in the situation of limited network resources.

**Remark 5**. From Table 2, comparing the different types of synchronization, we can detect that by changing the value of the projective factor α and Lipschitz parameters α_1_, α_2_, and α_3_, (for synchronization, we make α = 1, α_1_ = α_2_ = α_3_ = 0.01, for anti-synchronization, α = −1, α_1_ = α_2_ = 1, 000, α_3_ = 0.01), the control updating instants can be changed. It is clearly noticed that the updating frequency of the controller for synchronization is faster than anti-synchronization. That is to say, altering the values of parameters can not only affect the updating frequency.

**Table 2:** Performance comparison of periodic sampling control and proposed scheme.

**Agents**	**Node 1**			**Node 2**			**Node 3**		
**Dimension**	**1**	**2**	**3**	**1**	**2**	**3**	**1**	**2**	**3**
Traditional method	400	400	400	400	400	400	400	400	400
Our method (Syn)	369	364	338	389	381	378	191	262	261
Max time interval	0.7043	0.0849	0.0673	0.4968	0.5077	0.4985	2.3849	1.0406	1.2634
Mean time interval	0.0136	0.0137	0.0148	0.0129	0.0131	0.0262	0.0191	0.0132	0.0192
Traditional method	150	150	150	150	150	150	150	150	150
Our method (Anti-syn)	49	45	56	134	33	9	69	32	19
Max time interval	1.2503	1.4696	2.4314	2.3156	3.8352	0.1149	2.5068	3.0734	0.0912
Mean time interval	0.1020	0.1111	0.0893	0.0373	0.1515	0.5556	0.0725	0.1563	0.2632

## 5. Conclusion

A fresh event-triggered impulsive control scheme for a class of time-varying uncertain coupled MNNs has been proposed in this article. Considering the parameter mismatch and coupled structure of the proposed system, the event-triggered impulsive scheme has been constructed to solve the problem of projective quasi-synchronization. Accordingly, the established triggered functions with uncertainties and projective factors make the quasi-synchronization criteria more universal than conventional neural networks. Besides, the Zeno behavior can be naturally escaped through the design of proper triggered conditions.

Furthermore, the proposed mechanism has been employed to deal with projective quasi-synchronization, which can not only expand the types of synchronization but also avoid unnecessary energy consumption. As shown in the simulation, the designed self-triggered algorithm is reasonable in terms of avoiding Zeno behavior. However, just quasi-synchronization and quasi-anti-synchronization are considered, from the projective factor point of view, the time-varying factor is more universal for application, especially in social networks (Cheng et al., 2022), object/scene reconstruction (Tang et al., 2021), and 3D object recognition (Chen Y., 2022) etc. Therefore, in future study, the obtained scheme will be developed for projective quasi-synchronization with time-varying projective factors and more practical networks will be considered.

## Data availability statement

The original contributions presented in the study are included in the article/supplementary material, further inquiries can be directed to the corresponding author/s.

## Author contributions

MY designed the research and wrote the paper. MY and XL performed the research. JH contributed new reagents and analytic tools. JH and SW analyzed the data. All authors contributed to the article and approved the submitted version.

## Funding

This study was supported in part by the National Natural Science Foundation of China under Grants 62106020 and U1836106, the Fundamental Research Funds for the Central Universities under Grant FRF-IDRY-20-022, the China Postdoctoral Science Foundation under Grant 2021M690355, the Beijing Natural Science Foundation under Grants 19L2029 and L211020, the Scientific and Technological Innovation Foundation of Foshan under Grants BK20BF010 and BK21BF001, andd the Postdoctoral Research Foundation of Shunde Graduate School of University of Science and Technology Beijing under Grant 2021BH008.

## Conflict of interest

The authors declare that the research was conducted in the absence of any commercial or financial relationships that could be construed as a potential conflict of interest.

## Publisher's note

All claims expressed in this article are solely those of the authors and do not necessarily represent those of their affiliated organizations, or those of the publisher, the editors and the reviewers. Any product that may be evaluated in this article, or claim that may be made by its manufacturer, is not guaranteed or endorsed by the publisher.
